# Comprehensive evaluation of stool-based diagnostic methods and benzimidazole resistance markers to assess drug efficacy and detect the emergence of anthelmintic resistance: A Starworms study protocol

**DOI:** 10.1371/journal.pntd.0006912

**Published:** 2018-11-02

**Authors:** Johnny Vlaminck, Piet Cools, Marco Albonico, Shaali Ame, Mio Ayana, Jeffrey Bethony, Giuseppe Cringoli, Daniel Dana, Jennifer Keiser, Maria P. Maurelli, Antonio Montresor, Zeleke Mekonnen, Greg Mirams, Rodrigo Corrêa-Oliveira, Roger Prichard, Nour Rashwan, Laura Rinaldi, Somphou Sayasone, Eurion Thomas, Jaco J. Verweij, Jozef Vercruysse, Bruno Levecke

**Affiliations:** 1 Department of Virology, Parasitology and Immunology, Ghent University, Merelbeke, Belgium; 2 Center for Tropical Diseases, Sacro Cuore Don Calabria Hospital, Negrar, Italy; 3 Department of Life Sciences and Systems Biology, University of Turin, Turin, Italy; 4 Laboratory Division, Public Health Laboratory-Ivo de Carneri, Chake Chake, United Republic of Tanzania; 5 Institute of Health, Jimma University, Jimma, Ethiopia; 6 Laboratory of Microbiology, Immunology and Tropical Medicine, George Washington University Medical Center, Washington, DC, United States of America; 7 Department of Veterinary Medicine and Animal Productions, University of Naples Federico II, Naples, Italy; 8 Department of Medical Parasitology and Infection Biology, Swiss Tropical and Public Health Institute, Basel, Switzerland; 9 Department of Control of Neglected Tropical Diseases, World Health Organization, Geneva, Switzerland; 10 New Zealand division, Techion Group Ltd, Dunedin, New Zealand; 11 Laboratory of Molecular and Cellular Immunology, Research Center René Rachou - FIOCRUZ, Belo Horizonte, Brazil; 12 Institute of Parasitology, McGill University, Quebec, Canada; 13 National Institute of Public Health, Ministry of Health, Vientiane, Lao People’s Democratic Republic; 14 United Kingdom division, Techion Group Ltd, Aberystwyth, United Kingdom; 15 Laboratory for Medical Microbiology and Immunology, St. Elisabeth Hospital, Tilburg, The Netherlands; University of Cambridge, UNITED KINGDOM

## Abstract

**Background:**

To work towards reaching the WHO goal of eliminating soil-transmitted helminth (STH) infections as a public health problem, the total number of children receiving anthelmintic drugs has strongly increased over the past few years. However, as drug pressure levels rise, the development of anthelmintic drug resistance (AR) is more and more likely to appear. Currently, any global surveillance system to monitor drug efficacy and the emergence of possible AR is lacking. Consequently, it remains unclear to what extent the efficacy of drugs may have dropped and whether AR is already present. The overall aim of this study is to recommend the best diagnostic methods to monitor drug efficacy and molecular markers to assess the emergence of AR in STH control programs.

**Methods:**

A series of drug efficacy trials will be performed in four STH endemic countries with varying drug pressure (Ethiopia and Brazil: low drug pressure, Lao PDR: moderate drug pressure and Tanzania: high drug pressure). These trials are designed to assess the efficacy of a single oral dose of 400 mg albendazole (ALB) against STH infections in school-aged children (SAC) by microscopic (duplicate Kato-Katz thick smear, Mini-FLOTAC and FECPAK^G2^) and molecular stool-based diagnostic methods (quantitative PCR (qPCR)). Data will be collected on the cost of the materials used, as well as the time required to prepare and examine stool samples for the different diagnostic methods. Following qPCR, DNA samples will also be submitted for pyrosequencing to assess the presence and prevalence of single nucleotide polymorphisms (SNPs) in the β-tubulin gene. These SNPs are known to be linked to AR in animal STHs.

**Discussion:**

The results obtained by these trials will provide robust evidence regarding the cost-efficiency and diagnostic performance of the different stool-based diagnostic methods for the assessment of drug efficacy in control programs. The assessment of associations between the frequency of SNPs in the β-tubulin gene and the history of drug pressure and drug efficacy will allow the validation of these SNPs as a marker for AR in human STHs.

**Trial registration:**

The trial was retrospectively registered the 7^th^ of March 2018 on Clinicaltrials.gov (ID: NCT03465488).

## Author summary

Soil-transmitted helminths (STHs) affect 1.4 billion people worldwide and cause significant morbidity when the intensity of infection is high. Currently, these infections are controlled in school-aged children by preventive chemotherapy with the benzimidazole drugs albendazole (ALB) or mebendazole (MEB). However, for the success of these control programs, it is essential to keep track of the efficacy of these drugs and to screen parasite populations for a possible rise of anthelmintic resistance (AR). In this light, a series of trials will be performed to assess the efficacy of ALB treatment against STH in four endemic countries with varying drug pressure. Both microscopic and molecular stool-based diagnostic methods will be used to evaluate drug efficacy. DNA samples will also be analysed for the presence and prevalence of mutations in a gene that was previously linked to AR in animal STHs. The results of these trials will provide evidence on the efficacy of ALB, help select the optimal diagnostic method to assess drug efficacy and provide information regarding the usefulness of genetic markers for AR detection in human STHs.

## Introduction

Soil-transmitted helminths (STHs) include *Ascaris lumbricoides*, *Trichuris trichiura*, and two hookworm species, namely *Necator americanus* and *Ancylostoma duodenale*. Global estimates indicate that more than 1.4 billion people are infected with at least one of the four STH species [1]. They are responsible for an estimated 3.3 million disability-adjusted life years (DALYs) of which 1.2 million occurred in children which is the highest burden among all neglected tropical diseases (NTDs) [2]. Preventive chemotherapy (PC) is the main strategy to control the morbidity caused by STHs. This entails the periodic administration of a single, oral dose of albendazole (ALB; 400 mg) or mebendazole (MEB; 500 mg) mainly to preschool-aged (preSAC) and school-aged children (SAC) through a school-based PC programs [3]. However, as part of the control program for lymphatic filariasis—another NTD which share geographical distribution with STHs—ALB is also co-administered with ivermectin to the whole community [4, 5]. Powered by the London Declaration on NTDs, the global coverage of children in PC programs has increased from ~30% in 2011 to 63.6% in 2016 [6], and, if the goal of covering at least 75% of children by 2020 will be reached, over 900,000 DALYs will be averted [7]. The laudable long-term aim is to eliminate STHs as a public health problem, and to eventually declare targeted geographical areas free of infections [8].

However, this high level of drug pressure makes PC programs highly vulnerable to the development of anthelmintic resistance (AR). First, we are relying on two drugs (ALB and MEB) of the same class (benzimidazole (BZ) drugs), and with the same mode of action (the inhibition of the polymerization of microtubules). Hence the emergence of AR is likely to occur as drug donations expand. This has been substantiated in veterinary medicine [9–11], where AR has developed within a decade of the introduction of every anthelmintic class [12]. Moreover, the development of AR against one BZ drug would most likely be accompanied by poor anthelmintic drug efficacy of the other BZ drug. Second, drugs are administered in single doses and, although a single dose is operationally justified, it never achieves 100% efficacy [13–16]. Consequently, this practice may further select for the development of AR when suboptimal doses are widely applied over a significant period. in light of breaking transmission of soil-transmitted helminthiasis there is growing interest to move from a school-based towards a community-based approach, which will further reduce the worm population that is not exposed to drugs [17, 18]. Finally, few anthelmintic drugs are licensed for the treatment of STH infections in humans [19, 20]. Thus, should AR against BZ drugs eventually emerge and spread, PC-based control of STHs will be even more limited than at present with few acceptable alternative options. This re-enforces the urgent necessity for increased accessibility of anthelmintic drugs of different anthelmintic classes and thoroughly designed surveillance systems that allow for the detection of any changes in anthelmintic drug efficacy arising through the evolution of AR in these helminths.

In 2013, WHO developed a manual to evaluate drug efficacy (WHO 2013). And although a number of countries used this manual to evaluate the efficacy of the anthelminthic used in their programme, a systematic surveillance system to monitor drug efficacy and emergence of AR is lacking. One of the main reasons for this lack of monitoring systems is the absence of a framework that guides and supports health-care decision makers in planning, performing and reporting surveys. The development of such a framework is not straightforward [21]. Moreover, PC programs typically operate in resource-constrained settings, and therefore it is indispensable that health-care decision makers have some pliancy to minimize financial and technical resources, while assuring a reliable assessment of the drug efficacy and spread of AR [22–25]. Important obstacles to globally monitor patterns of changing drug efficacy and emergence of AR are (i) the need for diagnostic laboratories with experienced technicians to perform the surveys, and experienced staff to analyze and report the data; (ii) the absence of a quality assurance system that guarantees data of high quality; (iii) a validated marker linked to AR; (iv) on-site diagnostic methods that allow for early detection of AR, and (v) the lack of overall guidance in designing surveys to monitor drug efficacy in PC programs.

To conclude, AR is a real threat for PC programs targeting human STHs. To establish a surveillance system and to further ensure the efficacy of the administered drugs, there is a need for (i) diagnostic methods that effectively mitigate important obstacles of performing, analysing and reporting drug efficacy surveys in resource poor settings, and a validated molecular marker to detect emergence of AR at an early stage, (ii) a surveillance system that monitors global patterns of drug efficacy and AR and (iii) tools to plan routine AR monitoring, and to follow-up global changes in drug efficacy and spread of AR over time. Each of the aforementioned needs will be addressed in a separate work package of the Starworms project (STop Anthelmintic Resistant Worms; www.starworms.org). **The main objective** of the project is to **strengthen the monitoring** and **surveillance** of **drug efficacy** and **AR** in programs aimed at eliminating and controlling **STH infections** in humans.

This document describes the rationale, specific objectives and methods/design, and discusses the expected output and challenges for work package 1 of the Starworms trial which aimes to evaluate stool-based diagnostic methods to assess drug efficacy and BZ resistance markers to assess the emergence of AR. For more details on the other work packages, we refer to the project website [26].

### Introduction to work package 1

Currently, the reduction in number of eggs excreted in stool after drug administration (egg reduction rate, ERR) is the recommended method for monitoring the efficacy of anthelmintic drugs against STHs [27]. In contrast to other available assays, it allows for the assessment of the efficacy of any drug against all STHs [28–31].

Today, a variety of egg counting methods have been recommended by WHO for the assessment of drug efficacy [27], of which Kato-Katz thick smear is the most commonly applied. Egg counting still requires a minimum amount of laboratory equipment and experienced laboratory technicians to ensure quality of the data obtained [22, 24, 32]. Furthermore, it is essential that samples are analysed quickly after collection to ensure the visualisation of hookworm eggs and that results are adequately analysed, applying the most appropriate summary statistics [15, 27] and are then reported to the responsible authorities. There is often limited availability of laboratory capacity and a poor reporting of the results obtained. This prompts the need for an improved diagnostic method that enables (i) egg counts to be performed without the use of a microscope, (ii) automated egg counting, and (iii) submission of results from remote locations *via* the internet for quality control of egg counts, analysis and reporting. Such a method would eliminate the need for a microscope or highly skilled technicians/clinicians or administrators while providing and centralizing drug efficacy results.

A method that largely meets this target product profile is the FECPAK^G2^ platform. Recently, it has been launched as a complete remote-location diagnostic method for sheep/cattle farmers and their veterinarians to assess the intensity of helminth infections and efficacy of drugs [33]. The platform accumulates helminth eggs into one microscopic view after which digital images are taken [34]. Images are stored by the associated software and can be uploaded to a remote server when an Internet connection is available. Later, a web-based laboratory technician can count the eggs visible in the images, after which the results are returned to the user by e-mail. The FECPAK^G2^ platform thus potentially eliminates the need for skilled technicians on-site. The online software allows easy access for quality control of egg counting and the production of standardized analysis and reports. More importantly, it opens the door to automated egg counting by egg recognition software [35]. Recently, Ayana et al. (in press) [36] modified and optimized a FECPAK^G2^ protocol for the detection and quantification of human STH eggs in stool. However, it remains unclear whether it provides equivalent ERR results to those obtained by diagnostic methods recommended by WHO, and whether it would result in more efficient use of financial and technical resources under field conditions.

From a research perspective, there is a growing interest to apply quantitative polymerase chain reaction (qPCR) for the assessment of drug efficacy. In contrast to the egg counting methods, qPCR can differentiate the different hookworm species, and thus allow evaluation of BZ efficacy against the different hookworm species [37, 38]. This is of particular interest given that there could be differential susceptibility towards BZ drugs among hookworm species, and that there is increasing evidence of zoonotic transmission of *Ancylostoma ceylanicum* between dogs and humans [39–42]. Although studies have linked amount of DNA with egg output [37, 43], to date, qPCR has not yet been applied for the assessment of drug efficacy.

Early detection of BZ resistance is essential to anticipate the efficacy of drugs in STH control programs, as it is more difficult to mitigate the spread of AR when the initial frequency is already high [44]. However, both *in vivo* (e.g. ERR) and *in vitro* assays (e.g. egg hatch assay) lack the sensitivity to detect the emergence of AR at an early stage. For example, in animals ERR was only able to detect AR when the proportion of resistant STHs exceeded 25% [45], a proportion that may impede mitigating the spread of AR. A more sensitive alternative is a molecular test based on resistance-associated mutations in targeted genes. Due to the unfortunate and costly experiences of AR in veterinary medicine, BZ resistance has been researched in detail in animal STHs, providing strong evidence of molecular markers associated with BZ resistance. An overview of the markers for BZ resistance in animal STH is provided by Von Samson-Himmelstjerna et al., [46].

Generally, BZ resistance in animal STHs is caused by single nucleotide polymorphisms (SNPs) in the gene encoding β-tubulin at codons 167 (TTC to TAC), 198 (GAA to GCA) or 200 (TTC to TAC), but the relative association of these SNPs with BZ resistance varies considerably across animal STH species [47–49]. Various methodologies to detect and quantify these SNPs in animal STH have been developed through an international Consortium for Anthelmintic Resistance SNPs (CARS), [50] of which some have now also been developed and applied for human STHs [29, 30, 51–53]. The few studies assessing SNPs in human STHs suggest that (i) polymorphisms are predominantly found in codon 200, and (ii) that resistance-associated mutations increased after drug administration (seen for *T*. *trichiura*), (iii) but that there was no clear association with reduced drug efficacy. However, the results should be interpreted with caution. First, they were based on a small number of participants (ranging from 3 to 31 participants) across a limited number of geographical areas (Kenya, Haiti, Panama and Tanzania). Second, STH data were collected applying a variety of study methodologies. For example, the samples originated from a wide range spectrum of the population (pre-SAC, SAC and adults) and were examined applying different egg counting methods (Kato-Katz thick smear, McMaster egg counting method and FLOTAC), which makes comparison across studies difficult.

Most technologies to assess SNPs linked to BZ resistance are only available in well-equipped laboratories. Recently, Rashwan et al., [54] published a loop-mediated isothermal amplification (LAMP) assay for the assessment of β-tubulin polymorphisms in human STHs. Due to its simplicity, ruggedness and low cost, LAMP could provide major advantages in detecting AR on-site. However, the method needs to be further validated on field samples. Another strategy to further reduce the operational costs is to apply a pooled examination strategy. Examination of pooled stool has already proved to allow for reduction in technical and financial resources for other egg counting methods such as Kato-Katz thick smear [55].

### Study objectives

The overall aim of this study is to recommend the best diagnostic methods to monitor drug efficacy and status of AR in STH programs. These will then be applied in work package 2 (the establishment of a surveillance system to monitor the global patterns of drug efficacy and emergence of AR in STH programs). The specific objectives are to:

1. Assess equivalence of ERRs measured by Kato-Katz thick smear, Mini-FLOTAC and FECPAK^G2^
2. Assess the diagnostic performance of Kato-Katz thick smear, Mini-FLOTAC, FECPAK^G2^ and qPCR
3. Assess the costs linked with assessing drug efficacy by Kato-Katz thick smear, Mini-FLOTAC and FECPAK^G2^
4. Provide proof-of-principle for qPCR to assess intensity of infection and drug efficacy
5. Assess associations between frequency of SNPs linked to BZ resistance measured by pyrosequencing and history of drug pressure and drug efficacy
6. Compare pyrosequencing and LAMP for the assessment of SNPs linked to BZ resistance
7. Assess pooling samples as a cost-saving strategy to determine infection intensity (qPCR) and frequency of SNPs linked to BZ resistance (pyrosequencing and LAMP)

## Methods

### Ethics approval and consent to participate

The Starworms protocol has been reviewed and approved by the Institutional Review Board (IRB) of the faculty of medicine of Ghent University, Belgium (Ref. No B670201627755). The trial protocol was subsequently also reviewed and approved by the IRBs associated with each trial site (Ethical Review Board of Jimma University, Jimma, Ethiopia: RPGC/547/2016; National Ethics Committee for Health Research (NECHR), Vientiane, Lao PDR: 018/NECHR; Zanzibar Health Research Council, United Republic of Tanzania: ZAMREC/0002/February/2015 and the Institutional Review Board from Centro de Pesquisas René Rachou, Belo Horizonte, Brazil: 2.037.205).

Parent(s)/guardians of participants will sign an informed consent document indicating that they understand the purpose of and procedures required for the study and that they are willing to have their child participate in the study. If the child is ≥5 years, he/she has to orally assent in order to participate in the study. Participants of ≥12 years of age are only included if they sign an informed consent document indicating that they understand the purpose of the study and procedures required for the study and are willing to participate in the study.

### General design of the field trials

**Fig 1:**
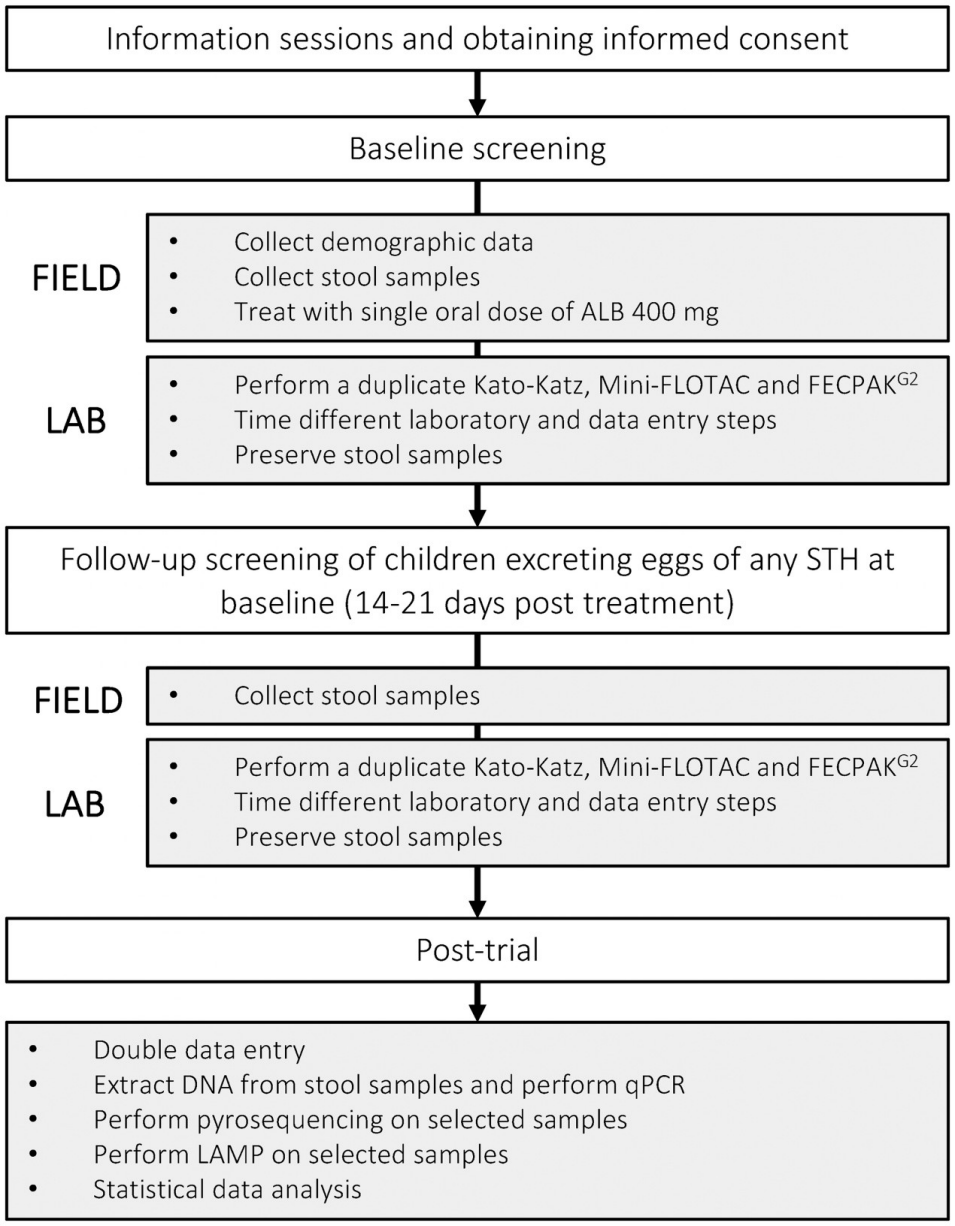
A schematic overview of the different steps of the field trials.

A series of drug efficacy trials will be performed in four STH endemic countries (Brazil, Ethiopia, Lao PDR and Tanzania). These trials will be designed to assess the efficacy of a single oral dose of 400 mg ALB against STH infections in SAC by a variety of stool-based diagnostic methods. At the start of each trial, schools will be visited by the local principal investigator (PI) and a team of field officers, who will explain the planned trial and sampling method to the parents and teachers and the children. At baseline, SAC will be asked to provide a fresh stool sample. All children that meet all inclusion criteria and none of the exclusion criteria (see Table 1) will be enrolled in the study. They will be treated with a single oral dose of 400 mg ALB under supervision. The ALB to be used in the different studies is manufactured by GlaxoSmithKline and donated to WHO and originates from the same production batch (Batch Nr: 335726). All collected stool samples will be processed to determine the fecal egg counts (FECs; expressed in eggs per gram of stool (EPG)) for each STH using Kato-Katz thick smear (single and duplicate), Mini-FLOTAC and FECPAK^G2^. During baseline evaluation, only stool samples found to contain at least 13 eggs on duplicate Kato-Katz or at least 15 eggs on Mini-FLOTAC for at least one of the three STHs will be preserved for further molecular analysis. Fourteen to 21 days after drug administration, a second stool sample will be collected from all the children that were excreting eggs of any STH at baseline based on Kato-Katz thick smear and Mini-FLOTAC. Stool samples will be examined by Kato-Katz thick smear (single and duplicate), Mini-FLOTAC and FECPAK^G2^. All follow-up samples will be preserved for further molecular analysis. To gain insights into the operational costs for assessing drug efficacy, the time to process stool with Kato-Katz thick smear, Mini-FLOTAC and FECPAK^G2^, and the time needed to enter data and to draft reports will be assessed. The different steps of the trials are schematized in Fig 1.

**Table 1:** Inclusion and exclusion criteria to be followed during the recruitment of participants for the field trials.

Inclusion criteria	Exclusion criteria
Subject is 5–14 years of age Subject is otherwise in healthy condition (based on medical history and physical examination) Parent(s)/guardian(s) of subject signed an informed consent document indicating that they understand the purpose of and procedures required for the study and that they are willing to have their child participate in the study Subject of ≥5 years has assented to participate in the study Subject of ≥12 years has signed an informed consent document indicating that they understand the purpose of the study and procedures required for the study, and are willing to participate in the study Subject has provided a stool sample of at least 9 grams.	Subject has active diarrhoea (defined as the passage of 3 or more loose or liquid stools per day) at baseline or follow-up. Subject has an acute medical condition or is experiencing a severe concurrent medical condition Subject has a known hypersensitivity to ALB or MEB Subject has received anthelmintic treatment within 90 days prior to the start of the treatment Subject vomited within 4 hours following drug ingestion. Subject is not able to provide a stool sample of min 9 grams at baseline or follow-up. Subject has not swallowed the entire tablet.

### Study population

The study will focus on SAC (age 5–14) since they are the major target of PC programs targeting STH, and they usually represent the group with highest worm burdens for *A*. *lumbricoides* and *T*. *trichiura* [56]. This study will be conducted in Ethiopia, Tanzania, Lao PDR and Brazil. The selection of these sites is based on their experience in assessing drug efficacy [13, 15], evaluating the performance of diagnostic methods [57–59] and the availability of well-equipped diagnostic facilities and skilled personnel, and PC history (Table 2).

**Table 2:** An overview of the countries, study sites and institutions involved in work package 1 of the Starworms project as well as their respective drug pressure and the type of benzimidazole drug administered.

Country	Study site	Institution	Drug Pressure *	Drug
**Brazil**	Americaniñas, Belo Horizonte	Fiocruz—Research institute of Renê Rachou	Low	ALB
**Ethiopia**	Jimma Town, Jimma Zone	Jimma University	Low	ALB
**Lao PDR**	Nam Bak, Luang Prabang	National institute of public health	Medium	MEB
**Tanzania**	Chake Chake, Pemba	Public Health Laboratory—Ivo de Carneri	High	ALB

* The classification of the countries into the four levels of drug pressure was based on the reported national coverage of drug administration to preschool (preSAC) and school aged children (SAC) for the last 5 years (2009–2014; Preventive Chemotherapy Database of the WHO). Very high drug pressure: coverage >80% for preSAC and SAC for each of the last 5 years; moderately high: coverage >50% for preSAC and/or SAC for each of the last 5 years; moderate: coverage >30% for preSAC or SAC for each of the last 5 years; low: coverage is <30% for both preSAC and SAC for each of the last 5 years.

### Sample size calculation

A sample size was calculated to test the alternative hypothesis that FECPAK^G2^, Mini-FLOTAC and single Kato-Katz thick smear provide equivalent drug efficacy results measured by ERRs compared to a duplicate Kato-Katz thick smear. Given the differences in drug efficacy of ALB across the STH species [15, 21] (*Ascaris*: ~99%, hookworms: ~96%, *Trichuris*: ~65%), a level of equivalence that is acceptable for *Trichuris* may not be acceptable for *Ascaris*. Therefore, a species-specific level of equivalence was used. For *Ascaris* the level of equivalence was set at 2.5-point percentage, for hookworms and *Trichuris* the level of equivalence was set at 5.0 and 10 point percentage, respectively. Both type I and II errors were set at 0.05.

To calculate the corresponding sample size for each of the STH species, we performed a simulation study. This simulation study considered (i) variation in ERR and baseline FECs across and within STH species, (ii) variation in FECs introduced by the egg counting process, (iii) the paired ERR results across egg count methods, and (iv) a post-hoc correction for a pair-wise comparison. Based on the simulation, at least 110, 100 and 12 complete cases are required for *Trichuris*, hookworm and *Ascaris*, respectively. A detailed description of the sample size calculation is available in the supplementary information (S1 Info).

### Laboratory procedures

We will provide participants with a container for the collection of stool samples. The samples will be collected and stored in cooler boxes with ice-packs, which will then be transported to the laboratory at the respective study site for further analysis. Upon arrival in the laboratory, samples will be thoroughly homogenized using a wooden spatula to obtain a more homogeneous distribution of STH eggs in the stool [60]. A detailed standard operating procedure (SOP) on sample homogenisation is available in the supplementary information (S2 Info).

### Egg counting methods

#### Kato-Katz thick smear

Kato-Katz thick smears will be prepared on microscope slides using a square template with a hole of 6 mm diameter and 1.5 mm deep, which is assumed to sample 41.7 mg of stool [61]. Two Kato-Katz thick smears will be prepared per sample (slide A and B) and to avoid clearing of hookworm eggs, all slides will be examined within 30–60 min for the presence of STH eggs. The number *A*. *lumbricoides*, *T*. *trichiura* and hookworm eggs will be counted and recorded per slide. Slide A will represent a single Kato-Katz thick smear. For the duplicate Kato-Katz thick smear, the egg counts of slide A and B are added and multiplied by 12 to obtain the FEC. A detailed SOP on how the duplicate Kato-Katz thick smears will be processed is provided in S3 Info.

#### Mini-FLOTAC method

The Mini-FLOTAC method will be performed as described earlier [62]. In short, the sample recipient of the Fill-FLOTAC will be filled with fresh stool. Subsequently, the stool will be thoroughly homogenized with 38 ml of flotation solution (FS) (saturated salt solution, density = 1.20). The suspension will then be filtered through the integrated Fill-FLOTAC filter, and transferred into the two chambers of the Mini-FLOTAC device. After 10 minutes, the top part of the chambers will be translated, and both chambers of the Mini-FLOTAC device will be screened for the presence of STH eggs under a light microscope using a 100x magnification. The number of eggs in both Mini-FLOTAC chambers combined will be counted and recorded for *A*. *lumbricoides*, *T*. *trichiura* and hookworm, separately. A detailed SOP on the Mini-FLOTAC method can be found in the supplementary information (S4 Info).

#### FECPAK^G2^ method and automated egg counting

Ayana et al. (in press) [36] describes the FECPAK^G2^ method for counting STH eggs in human stool. Stool analysis starts by weighing 3 grams of fresh stool in a Fill-FLOTAC. Subsequently, the stool will be homogenized in tap water. Afterwards, the stool suspension will be transferred to the FECPAK^G2^ sedimenter and additional tap water will be added to the sedimenter (approximately 200 ml). The sedimenter will be inverted 3 times to mix the solution, after which it will be placed on an even surface at room temperature to allow STH eggs to sediment. The next day, the supernatant will be poured off and 80 ml FS will be added to the remaining slurry (15 ml) that is being retained in the bottom of the sedimenter by the plastic dam. The whole content of the sedimenter will then be poured into a FECPAK^G2^ filtration unit. The closed FECPAK^G2^ filtration unit will be inverted three times before transferring 455 μl of the suspension into each of the 2 wells of the cassette. Between each sampling, the cylinders will be inverted three times to homogenize the solution. After filling the wells, the cassette will be placed on a horizontal surface for a minimum of 20 minutes to allow accumulation of the STH eggs around the tip of the rod that is present in the centre of each well of the FECPAK^G2^ cassette. Subsequently, the cassette will be placed in a Micro-I device for image capture, which will be connected to a laptop or desktop computer. The device automatically images both wells. The digital images of the wells will be stored on the laptop / computer and will be uploaded to the main FECPAK^G2^ server when an Internet connection is available. After the imaging step, the cassettes will be emptied and rinsed with tap water for reuse. To count the eggs, sample images and details (sample ID, sample collection date, study site, etc.) will be uploaded from the server. Finally, the mark-up technician will identify and count any STH eggs present in the images using specialized software developed for this purpose. Results of the mark-up will be saved and stored automatically for reporting and analysis. Detailed SOPs on how to perform the FECPAK^G2^ and to mark-up the images are available in S5 Info. Stored images will also be submitted for analysis by specialized image processing software originally designed to detect and quantify helminth eggs in waste water [35].

#### Quality control egg counting procedure

A predefined, randomly selected subset of samples (10% of the total number of samples) will be re-evaluated by each of the three egg count methods. To this end, a senior researcher, who is blinded to the initial FECs, will re-count STH eggs across all three egg count methods. A third examiner will re-count STH eggs in case of discrepancies. Discrepancies are defined as (i) false negatives/positives, (ii) difference in egg counts >10 when the total number of eggs counted ≤100 or (iii) difference in egg counts >20% when more than 100 eggs are counted [24]. A detailed SOP on the performance of quality control for the egg counting methods is provided in the supplementary information (S6 Info).

### DNA-based methods

#### Stool preservation and DNA extraction

After performing the egg counting methods, stool samples will be preserved. At baseline, only those samples in which a sufficient number of eggs are counted for at least one STH species will be preserved (duplicate Kato-Katz thick smear: 13 eggs; Mini-FLOTAC: 15 eggs). At follow-up, all samples will be preserved, and this regardless of the number of STH eggs found. For each sample, 2 subsamples of 0.5 g will be preserved in 1 ml of absolute ethanol. A detailed SOP on sample preservation is provided in the supplementary information (S7 Info). Preserved samples will be stored in sample boxes at room temperature and shipped to Belgium for DNA extraction and subsequent molecular analysis. The DNA extraction protocol will be run on the automated QiaSymphony using a preceding mechanical lysis step by means of bead beating. This DNA protocol maximizes STH DNA yield from stool (Ayana et al, in preparation). DNA extraction will be performed at Ghent University (Belgium) and Elizabeth Tweesteden Hospital (The Netherlands). S8 Info provides a detailed SOP on DNA extraction.

#### qPCR

All DNA extracts will be analyzed for the presence of *A*. *lumbricoides*, *T*. *trichiura*, *N*. *americanus* and *A*. *duodenale* DNA using qPCR assays previously described [37, 63, 64]. S9 Info provides a detailed SOP on how we will perform the qPCR. The output of the qPCR analysis will be expressed in genome equivalents (GE) per ml DNA extract.

#### Pyrosequencing

In two subsets of the DNA samples, we will apply a previously described pyrosequencing protocol [30, 52] to determine frequency of mutant SNPs in the three codons of the ß-tubulin associated with BZ resistance. The first subset will consist only of baseline DNA samples, and will be used to assess the association between drug pressure history and the corresponding frequency of mutant SNPs linked to BZ resistance. Per study site, 60 baseline DNA samples positive for each STH species (*Ascaris*, *Trichuris* and hookworm) will be randomly selected from the available baseline DNA samples. To avoid these samples representing one school or one level of individual response to ALB, selection of the samples will be proportionately to the total number of cases per school and individual drug response (Table 3).

**Table 3:** Classification of the individual response to a single oral dose 400 mg ALB against STH based on the individual egg reduction rate (iERR).

STH	Cured	Responder	Poor responder	Non-responder
***Ascaris***	iERR = 100%	iERR ≥ 95%	85% ≤ iERR < 95%	iERR < 85%
***Trichuris***	iERR = 100%	iERR ≥ 50%	40% ≤ iERR < 50%	iERR < 40%
**Hookworms**	iERR = 100%	iERR ≥ 90%	80% ≤ iERR < 90%	iERR < 80%

We will use a second set of DNA samples to assess the association between individual responses to ALB by pyrosequencing the ß-tubulin gene. At each site, samples from at least 30 participants who were cured (baseline samples only) and from at least 30 participants who were still excreting eggs at follow-up (both baseline and follow-up samples) will be selected for each STH species separately. Participants which still excreted eggs will be stratified, into ‘responders’, ‘poor responders’ and ‘non-responders’. This stratification will be based on WHO thresholds used for classifying the efficacy into ‘satisfactory’ (‘responders’), ‘doubtful’ (‘poor responders’) and ‘reduced’ (‘non-responders’) [27] (Table 3). A total of 10 participants will be randomly selected for each stratum. Total DNA will be extracted from these stool samples and sent for pyrosequencing at McGill University (Canada).

#### LAMP

A previously described LAMP protocol [65] will be applied on a subset of 300 DNA samples that are withheld for pyrosequencing. The selection of these samples will be based on the frequency of SNPs linked to AR. Based on the pyrosequencing results, samples will be classified into four levels of SNP frequency, including absence of SNP linked to BZ resistance, and low, moderate and high frequency of SNPs linked to BZ resistance. The thresholds used to define the last three levels of frequency of SNPs will be based on the 1^st^ and 3^rd^ quartile of the frequency of SNP across all the samples, in which SNP linked to BZ resistance are present. Samples in which the frequency of a SNP for BZ resistance is higher than 0% but lower than the 1^st^ quartile will be classified into the low frequency group, samples in which the frequency of a SNP for BZ resistance is at least equal to the 3^rd^ quartile will be classified into the high frequency group. All other samples, in which SNPs linked to BZ resistance are found, will be classified into the moderate frequency group. For each STH species (*Ascaris*, *Trichuris*, and hookworms), 25 samples in each level of SNP frequency will be randomly selected and processed with the LAMP assay. The LAMP will be performed at McGill University (Canada) and at two study sites (Ethiopia and Tanzania).

#### Pooling stool samples for DNA-based diagnostic methods

For both qPCR and pyrosequencing/LAMP, pooled samples will be analyzed. For qPCR, aliquots of all stool samples preserved for further molecular analysis will be pooled into pools of 10, 20 and 60 individual samples. The procedure to pool stool was adapted from Mekonnen et al., [66]. A detailed SOP on sample pooling is available in supplementary information (S10 Info). For pyrosequencing and LAMP, only samples that are withheld for pyrosequencing will be pooled into pools of 10 individual samples. All downstream processes (DNA extraction extracted, qPCR and pyrosequencing/LAMP) will be performed as described in aforementioned sections.

### Cost analysis of the different diagnostic methods

To make objective estimates of the costs associated with the different diagnostic methods, we will collect data on the cost of the materials used as well as the time required to prepare and examine stool samples for the different stool-based diagnostic methods.

### Assessing costs of reagents and materials

Detailed pricing information for the list of materials needed to perform Kato-Katz thick smear and Mini-FLOTAC will be obtained from the local PI. The costs for the materials needed for the FECPAK^G2^ method will be obtained from the manufacturer (Techion Group Ltd.) and is expected to be the same for all four study sites. A list of materials included in the cost calculation for the Kato-Katz thick smear and Mini-FLOTAC method is provided in S11 Info. The costs associated with molecular analysis of the samples will include the cost for preservation and shipment of the stool samples as well as the laboratory materials used to perform DNA extractions and qPCR.

### Timing of diagnostic methods

Timers will be provided to each laboratory technician to measure the time needed to perform the different steps of each of the diagnostic methods. For all egg counting methods, the time taken to complete each step will be measured. In addition, the time needed for data entry and drafting a report will be recorded. The protocols of the egg counting methods include more details on the different timing steps (S3–S5, S8 and S10 Info).

### Data management

All data collected during the studies will be recorded in specifically designed record forms (S12 Info). The original results written down on the paper documents will be scanned and stored digitally. This data will later be entered into customized Excel-files by two separate individuals (S13–S15 Info). After completion of the data entry, the files of both data entry clerks will be compared for discrepancies (S16 Info). Potential mismatches will be highlighted and the true values verified using the scanned original record form.

### Study management and coordination

The PIs from each site are responsible for acquiring ethical approval by their local Institutional Review Board (IRB) and conducting the trial procedures as described in the original study protocol and study SOPs. The studies in the different countries will be coordinated by the project PI and the Starworms Coordinating Team at Ghent University. One week prior to the start of each study, a member of the Starworms Coordinating Team will visit the study site. Local team members will be informed on the study design, familiarised with the different study documents and receive both theoretical and practical training on how to perform different egg counting methods according to the Starworms SOPs. The Starworms staff member will remain on-site for at least 7 days after the start of the trial to ensure SOP adherence and to solve any issues that might arise.

### Statistical data analysis

After data collection and quality control, all data will be combined in a final, protected dataset for statistical analysis. After finishing work package 1, this dataset will be made available on the Starworms website (www.starworms.org). All statistical analysis will be performed in R (R Development Core Team, 2016).

### Assessment of the impact of previously conducted PC in reducing STH morbidity

We will evaluate both the prevalence and intensity of each different STH species in each of the four different study sites. The percentage of individuals included in the different classes of infection intensity will be reported in order to assess the efficacy of the PC intervention conducted until then in eliminating STH infection of moderate and heavy intensity.

### Assessment of the equivalence of egg reduction rates measured by Kato-Katz thick smear, Mini-FLOTAC and FECPAK^G2^

We will report the efficacy of a single oral dose of 400 mg ALB separately for *A*. *lumbricoides*, *T*. *trichiura* and hookworms, and for each FEC method by means of ERR, using the formula below:

ERR=100%×(arithmeticmean(FECatbaseline)−arithmeticmean(FECatfollow-up))/(arithmeticmean(FECatbaseline))

A bootstrap analysis will be used to determine the corresponding 95% confidence intervals (95% CI) for each egg counting method and for the pair-wise differences in ERR across diagnostic methods. A permutation test will be used to assess the equivalence in ERR between a duplicate Kato-Katz thick smear, and a single Kato-Katz thick smear, Mini-FLOTAC and FECPAK^G2^. Tukey’s method will be applied for multiple comparison between methods. In addition, the agreement between a duplicate Kato-Katz thick smear and the other egg counting methods in the assignment of drug efficacy into ‘satisfactory’, ‘doubtful’ and ‘reduced’ will be evaluated by Fleiss’ kappa statistic (κ_Fleiss_). This classification of the drug efficacy will be based on the criteria recently proposed by the WHO (Table 4) [27]. The value of this statistic indicates a slight (κ_Fleiss_ <0.2), fair (0.2 ≤ κ_Fleiss_ <0.4), moderate (0.4 ≤ κ_Fleiss_ <0.6), substantial (0.6 ≤ κ_Fleiss_ <0.8) and an almost perfect agreement (κ_Fleiss_ ≥ 0.8).

**Table 4:** Classification of the efficacy of a single oral dose 400 mg albendazole against STH.

STH	Satisfactory	Doubtful	Reduced
***Ascaris***	ERR ≥ 95%	85% ≤ ERR < 95%	ERR < 85%
***Trichuris***	ERR ≥ 50%	40% ≤ ERR < 50%	ERR < 40%
**Hookworms**	ERR ≥ 90%	80% ≤ ERR < 90%	ERR < 80%

### Assess the diagnostic performance of Kato-Katz thick smear, Mini-FLOTAC, FECPAK^G2^ and qPCR

For each diagnostic method, the sensitivity will be calculated using the combined results of the four methods (duplicate Kato-Katz thick smear, Mini-FLOTAC, FECPAK^G2^ and qPCR) as the diagnostic ‘gold’ standard. This means that a sample is considered positive if it tested positive on at least one of the four methods. The species specificity of all methods will be set at 100%, as indicated by the morphology of the eggs or by the species-specific primer sets used in qPCR. The corresponding 95% CI for sensitivity will be calculated separately for each diagnostic method. Differences in sensitivity between methods will be assessed by a permutation test (10,000 iterations). Tukey’s method will be applied for pair-wise comparisons between the methods. The impact of infection intensity on the sensitivity within each method will be explored by a logistic regression model, which will be fitted for each of the methods with their test result (positive/negative) as the outcome and the natural log transformed mean FEC across the three methods as covariate. The predictive power of the final models will be evaluated by the proportion of the observed outcome that was correctly predicted by the model. To this end, an individual probability >0.5 will be set as a positive test result, and negative if different. Finally, the sensitivity for each of the pre-defined values of FEC will be calculated based on these models.

### Intensity of infection

For egg counting methods, the agreement in egg counts will be evaluated by permutation tests (10,000 iterations) based on (i) Pearson’s correlation coefficient and (ii) the differences in FECs. Tukey’s method will be applied for pair-wise comparisons within each STH species. For qPCR, the agreement in GE numbers and FECs obtained by duplicate Kato-Katz thick smear will be evaluated by permutation tests (10,000 iterations) based on Pearson’s correlation coefficient.

Samples containing helminth eggs will be classified into low, moderate, and heavy infection intensity according to FECs obtained by duplicate Kato-Katz and following the thresholds proposed by WHO; for *A*. *lumbricoides* these are 1–4,999 EPG, 5,000–49,999 EPG, and >49,999 EPG; for *T*. *trichiura* these are 1–999 EPG, 1000–9,999 EPG, and >9,999 EPG; and for hookworm these are 1–1,999 EPG, 2,000–3,999 EPG and >3,999 EPG (WHO, 1998). For each of the other diagnostic methods, a receiver operating characteristic analysis will be performed to determine the corresponding FECs / GE numbers that allow classification of the infections into low, moderate and heavy using duplicate Kato-Katz thick smear as a reference. Finally, the proportion samples correctly classified as having a low, moderately and heavy infection intensity will be assessed separately for single Kato-Katz thick smear, Mini-FLOTAC, FECPAK^G2^ and qPCR.

### Estimate the costs linked to the assessment of drug efficacy by Kato-Katz thick smear, Mini-FLOTAC and FECPAK^G2^

The mean time to process and examine one stool sample will be calculated for each separate egg counting method. We will also calculate the mean time needed to enter the demographic data and test results of a single subject, and the time to analyze and report the data. To reach an estimation of the total cost associated per sample, we will combine these data with the estimation of the costs of the raw materials and labour time needed to analyze a sample by each of the different methods. To calculate the costs for analysing samples a fixed rate will be used. Finally, a one-way sensitivity analysis will be performed to assess the impact of each parameter on the total cost for each diagnostic method separately.

### Provide proof-of-principle for qPCR to assess infection intensity and drug efficacy by means of reduction in GE

The efficacy of a single oral dose of 400 mg ALB will be reported separately for *A*. *lumbricoides*, *T*. *trichiura* and hookworms by means of genome equivalent reduction rate (GERR), using the formula below: GERR = 100% x (arithmetic mean(GE at baseline)–arithmetic mean (GE at follow-up)) / (arithmetic mean (GE at baseline)).

The corresponding 95% CI for GERR will be determined by bootstrap analysis (10,000 iterations). A permutation test will be used to assess the equivalence in drug efficacy between a duplicate Kato-Katz thick smear by means of ERR and qPCR by means of GERR for each of the STH species separately. The same level of equivalence will be applied as described in section 4.8.1. In addition, the agreement between a duplicate Kato-Katz thick smear and qPCR in the assignment of drug efficacy into ‘satisfactory’, ‘doubtful’ and ‘reduced’ will be evaluated by κ_Fleiss_ statistic.

### Assess associations between frequency of SNPs linked to BZ resistance measured by pyrosequencing and history of drug pressure and drug efficacy

To assess the association between mutant SNPs linked to BZ resistance and history of drug pressure, a permutation test will be applied to evaluate differences in frequency of mutant SNPs linked to BZ in the baseline samples across the study sites. Tukey’s method will be applied for pair-wise comparisons between the study sites.

To assess the association between mutant SNPs linked to BZ resistance and drug efficacy, a permutation test will be applied to evaluate any difference in frequency of mutant SNPs linked to BZ across the four levels of individual response to ALB (Table 3). Tukey’s method will be applied for pair-wise comparisons between the four levels of individual response.

### Compare pyrosequencing and LAMP for the assessment of SNPs linked to BZ resistance

The sensitivity of the LAMP method to detect mutant SNPs linked to BZ resistance will be evaluated using the pyrosequencing method as the gold standard. The impact of SNP frequency on the sensitivity of LAMP will be explored by a logistic regression model with the LAMP result as outcome (negative/positive) and the frequency of SNPs linked to BZ resistance as covariate. The predictive power of the final models will be evaluated by the proportion of the observed outcome that was correctly predicted by the model. To this end, an individual probability >0.5 will be set as a positive test result, and negative in all other cases. The sensitivity for each of the pre-defined values of FEC will be calculated based on these models.

Finally, the κ-statistic will be determined to assess the agreement in the LAMP test result across the different study sites, Canada (McGill University), Ethiopia (Jimma University) and Tanzania (Public Health Laboratory—Ivo de Carneri)). The value of this statistic indicates a slight (κ <0.2), fair (0.2 ≤ κ <0.4), moderate (0.4 ≤ κ <0.6), substantial (0.6 ≤ κ <0.8) and an almost perfect agreement (κ ≥ 0.8).

### Assess pooling samples as a cost-saving strategy to determine infection intensity (qPCR) and occurrence of SNPs linked to BZ resistance (pyrosequencing and LAMP)

For qPCR, we will apply the Pearson’s correlation coefficient to assess the agreement between the mean frequency of GE number based on the examination of individual samples and the GE based on the examination of pooled samples will be evaluated. In addition, a permutation test (10,000 iterations) will be applied to test for differences in mean GE number between the examination of individual and pooled samples.

For pyrosequencing, the agreement between the mean frequency of SNPs based on the examination of individual samples and the frequency of SNPs based on the examination of pooled samples will be evaluated by the Pearson’s correlation coefficient. In addition, a permutation test will be applied to test for differences in mean frequencies between examination of individual and pooled samples.

For LAMP, the κ-statistic will be determined to assess the agreement in the LAMP test results obtained by an individual and a pooled examination strategy. The impact of SNP frequency on the sensitivity of LAMP will be explored by a logistic regression model with the LAMP result as outcome (negative/positive) and the frequency of SNPs linked to BZ resistance as covariate. The predictive power of the final models will be evaluated by the proportion of the observed outcome that was correctly predicted by the model. To this end, an individual probability >0.5 will be set as a positive test result, and negative in all other cases. The sensitivity for each of the pre-defined values of SNP frequency will be calculated based on these models.

## Discussion

The potential development of AR is a real threat for PC programs targeting STHs. It is facilitated by the ever-increasing quantity of anthelmintic drugs reaching at risk populations, the administration of drugs with the same mode of action at a suboptimal dose and the lack of alternative treatment options should AR eventually emerge and spread. It is therefore of paramount importance that the efficacy of the administered BZ drugs is regularly monitored. However, in order to establish a robust surveillance system to monitor drug efficacy there is a need for diagnostic methods that effectively mitigate important obstacles of performing, analysing and reporting drug efficacy surveys in resource poor settings, and a validated molecular marker to detect emergence of AR at an early stage. Based on the results from this study within the Starworms project, we aim to recommend diagnostic methods to monitor drug efficacy and molecular markers to assess the status of AR in STH control programs.

We will be the first to report a comprehensive comparison of two novel egg counting methods (Mini-FLOTAC and FECPAK^G2^) and DNA-based methods (qPCR) with the current WHO diagnostic standard (Kato-Katz thick smear). We will not limit our comparison to the diagnostic performance but expand our analysis to compare them for the intended use-case (assessment of drug efficacy by means of egg reduction rate) and consider the associated costs and time required to perform each of the different methods.

The inclusion of FECPAK^G2^ may create a game-changing shift in how drug efficacy can be monitored at a global scale. The online connectivity of FECPAK^G2^ allows easy access for quality control of egg counts and the production of standardized analysis and reports. It also opens the door to automated egg counting by egg recognition software, which when successful, could further increase throughput, reduce personnel costs and variation in egg counts between technicians or laboratories. We will provide proof-of-principle for qPCR to assess infection intensity and drug efficacy by means of GE numbers. Inclusion of qPCR will also allow for more accurate estimates of the sensitivity of the different diagnostic tests. This is of particular importance since there is no gold standard diagnostic method for STHs [67].

By standardizing the drug efficacy trials (e.g., egg count method, follow-up period, anthelmintic drug and statistical analysis) and strategically selecting the study sites (varying drug pressure history, and hence possibly differences in drug efficacy), we hope to provide complementary insights into SNPs in the β-tubulin gene as a marker for BZ resistance in human STHs. If these SNPs play a role in AR, we expect an increase in frequency in mutant SNPs in STHs as a function of increased drug pressure history and decreased individual response to treatment. However, this molecular part of the study may face some challenges, which may impede a straightforward interpretation of the results. First, if the expected trends in SNPs are not detected, it is not possible to completely exclude them as a marker for BZ resistance. Furthermore, it could also indicate that resistance to BZ has not yet emerged, which would also jeopardize any downstream objectives (e.g. comparison of pyrosequencing and LAMP for the assessment of SNPs linked to BZ resistance and assessing pooling samples as a cost-saving strategy to determine the frequency of SNPs linked to BZ resistance). It is also important to note that any observed trends would only suggest an association rather than causal relationship. Second, it remains unclear whether we will be able to select the number of cases per level of individual response for each of the different STH species. Finally, the classification of the individual response is based on the outcome of imperfect tests (sub-optimal sensitivity), further complicating the interpretation.

To conclude, the combined results of this study are expected to answer a number of outstanding questions with regards to how to best monitor drug efficacy and measure AR in populations that are being subjected to PC for STH infections. The results could be translated into novel recommendations for the scientific community on what is the best diagnostic method to assess drug efficacy in ongoing control programs and will create the basis to set up a robust surveillance system.

## Supporting information

S1 Info A detailed description of the sample size calculation. (PDF)

S2 Info A detailed SOP on sample homogenization. (PDF)

S3 Info A detailed SOP on how to perform the Kato-Katz thick smear method. (PDF)

S4 Info A detailed SOP on how to prepare flotation solution and how to perform the Mini-FLOTAC method. (PDF)

S5 Info A detailed SOPs on how to perform the FECPAK^G2^ method and how to perform the mark-up of the obtained images. (PDF)

S6 Info A SOP on how to perform and evaluate quality control of the stool-based microscopic methods. (PDF)

S7 Info A detailed SOP for sample preservation. (PDF)

S8 Info A detailed SOP on the extraction of DNA from preserved stool samples. (PDF)

S9 Info A detailed SOP on how to perform qPCR for the detection of STH species in DNA extract from stool samples. (PDF)

S10 Info A detailed SOP on sample pooling. (PDF)

S11 Info A list of materials included in the cost calculation for the Kato-Katz thick smear and Mini-FLOTAC method. (XLSX)

S12 Info The specific record forms for these trials. (PDF)

S13 Info Data entry form 1 (DEF01): Excel file to record demographic data, Kato-Katz and Mini-FLOTAC results during baseline and follow-up screening. (XLSX)

S14 Info Data entry form 2 (DEF02): Excel file to record the time it takes to enter baseline demographic data, coproscopic data obtained by Kato-Katz and to analyze and report the results. (XLSX)

S15 Info Data entry form 3 (DEF03): Excel file to record the time it takes to perform the different steps of sample preparation for the different coprological techniques. (XLSX)

S16 Info A detailed SOP on how to screen the data entry files of two separate data entry clerks for mismatches and errors. (PDF)
