# Supplementary material for: Comprehensive evaluation of stool-based diagnostic methods and benzimidazole resistance markers to assess drug efficacy and detect the emergence of anthelmintic resistance: A Starworms study protocol
Source: PLoS Negl Trop Dis. 2018 Nov 2;12(11):e0006912. doi: 10.1371/journal.pntd.0006912 (PMC6235403; doi:10.1371/journal.pntd.0006912)

Sample Size Calculation

A sample size was calculated to test the alternative hypothesis that FECPAK^{G2}, Mini-FLOTAC and single Kato-Katz thick smear provide equivalent drug efficacy results measured by egg reduction rates (ERR) compared to a duplicate Kato-Katz thick smear. The sample calculation for this pair-wise equivalence test is not trivial. First, the variance of ERR decreases as a function of increasing drug efficacy (Levecke et al., 2015), and since the efficacy of ALB significantly varies across the different STH species (*A. lumbricoides*: 99%; hookworms: 96.2%; *T. trichiura*: 63%; Levecke et al., 2014), one will need to determine the required sample size for each of the different STH species separately. Moreover, given this wide range in drug efficacy, one will also need to adjust the level of equivalence according to STH species; this is because a level of equivalence that is acceptable for *T. trichiura* may not be acceptable for *A. lumbriocides*. Rather than assessing equivalence of two drugs, we are assessing the efficacy of the same drug applying different diagnostic techniques on the same individuals, and hence observations are not independent. Due to the aforementioned challenges we performed a simulation study to determine the sample size for each of the three STH species separately.

Generally, this simulation consisted of three consecutive steps. First, data were generated by Monte Carlo simulation modifying the methodology described by Levecke et al. (2012). First, the distribution of parasites within the population before treatment was defined by a negative binomial distribution. This distribution is determined by two parameters: the mean fecal egg counts (FEC; expressed in eggs per gram of stool (EPG)) and aggregation of infections across animals (k). Low values of k indicate that only few individuals are excreting the majority of the eggs, high values indicate that egg counts are more equally distributed across the host population. From

this pre-defined distribution, 10,000 of FECs, each representing on individual, were randomly drawn. The observed FECs at baseline, however, will be different from the true baseline FECs due to the variation introduced by sampling eggs associated with the diagnostic technique. This component of variation was simulated using the Poisson distribution defined by the expected number of eggs counted (i.e. true baseline FEC/amount of stool (in grams) examined). Subsequently, a subset of *N* individuals was randomly drawn from all individuals found to be excreting eggs at baseline (observed baseline FEC >0 on at least 1 diagnostic technique). The true FEC at follow-up were generated from the Poisson distribution with mean equal to the true baseline FEC multiplied by *1-true drug efficacy*. The observed follow FECs were generated as described above for the baseline FECs. Subsequently, the difference in ERRs between diagnostic techniques and its corresponding confidence interval were determined. The ERR was calculated as described in the formula below, and is based on the arithmetic mean of baseline and follow-up FEC of the same individuals. The confidence interval was determined by applying a bootstrap analysis (5,000 iterations).

$$\text{ERR} = 1 - \frac{\text{arthmetic mean (follow-up FECs)}}{\text{arthmetic mean (baseline FECs)}}$$

Second, this data generating process was repeated for 1,000 times, and it was determined in how many cases the alternative hypothesis could not be rejected (the confidence interval included either the lower (*-d*) or upper limit (*+d)* of equivalence; type II error). Finally, the two previous steps were performed for different sample sizes (from 80 to 130 individuals).

For each three STH species different values for true drug efficacy, mean FEC, *k* and *d* were applied. Each of these values is represented in Table A. The true drug efficacy and mean FECs represent the lowest values reported in a multicenter drug efficacy trial designed to assess the efficacy of a single oral dose of ALB for the different STH species (Vercruysse et al., 2011). The lowest values were chosen as it is expected that variation in difference in ERR between techniques would increase as a function of decreasing drug efficacy (see above) and mean FEC (Levecke et al., under review). The parameter *k* was estimated as a function of the mean FEC (Levecke et al., 2015). The parameter *d* was set at 0.025 for *A. lumbricoides*, 0.05 for hookworms and 0.1 for *T. trichiura*.

Table A. The true drug efficacy, mean FEC, *k* and *d* for each of the three STH species.

	A. lumbricoides	***T. trichiura***	**Hookworm**
**True drug efficacy**	0.988	0.392	0.871
**Mean FEC (EPG)**	4,279	420	205
k	0.186	0.137	0.090
d	0.025	0.10	0.05

For simplicity, we only considered two techniques in the present study, but accounted for multiple pairwise comparison by setting the type I error at 0.017 (= 0.05 / 3 pairwise comparisons). The diagnostic techniques included were duplicate Kato-Katz (0.0417 g of stool) and FECPAK^{G2} (0.0294 g). These techniques were chosen based on a pilot study, indicating that this combination requires the highest sample size.

Figure A illustrates the change in power (= 1- type II error) as a function of sample size for *T. trichiura* and hookworms. When aiming for a power of at least 0.95, we need to have at least 110 complete cases for *T. trchiura*, and 100 for hookworms. For *A. lumbricoides* a sample size of 12 is required.

Figure A. The poweer (1- type II error) as a function of sample size for *T. trichiura* (black line) and hookworms (red line). The dashed vertical lines represent the sample size that corresponds with a power of at least 0.95, the dashed horizontal line represents a power of 0.95.

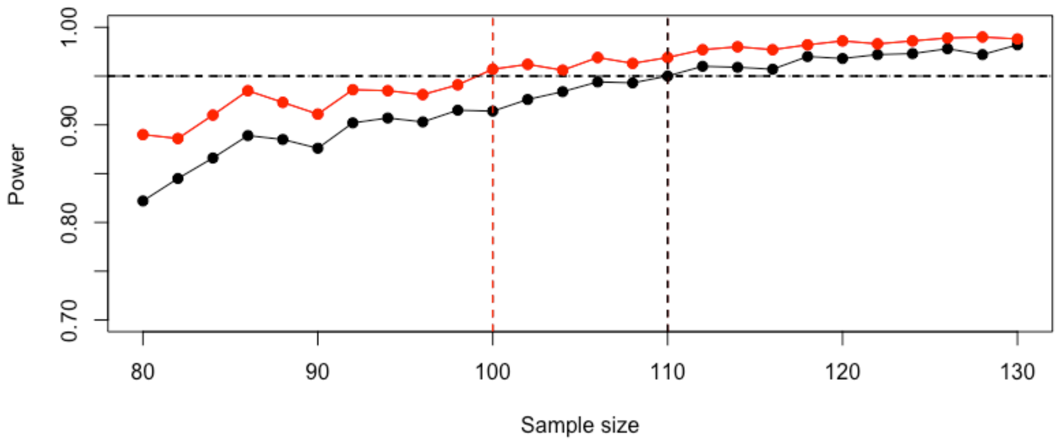

Supplement: S1 Info — (PDF) [file pntd.0006912.s001.pdf]
