# Supplementary material for: Comprehensive evaluation of stool-based diagnostic methods and benzimidazole resistance markers to assess drug efficacy and detect the emergence of anthelmintic resistance: A Starworms study protocol
Source: PLoS Negl Trop Dis. 2018 Nov 2;12(11):e0006912. doi: 10.1371/journal.pntd.0006912 (PMC6235403; doi:10.1371/journal.pntd.0006912)
Supplement: S2 Info — (PDF) [file pntd.0006912.s002.pdf]

Stool homogenization.

1. Purpose

This SOP describes the procedure on how to homogenize the stool samples before moving on to starting with the preparation of the different diagnostic techniques (i.e. duplicate Kato-Katz, Mini-FLOTAC and FECPAK^{G2}).

A crucial aspect of the starworms study is the timing of the different diagnostic procedures. Therefore, we will organize all samples in batches of 10, and we will record the time required to prepare all samples in each batch by the different techniques (Kato-Katz, Mini-FLOTAC and FECPAK^{G2}).

2. Equipment

- Spatula or wooden tongue depressor

3. Procedures

1. Homogenize a batch of stool samples by stirring each stool sample thoroughly using a spatula or wooden tongue depressor until they are fully homogenized: the color, consistency and content of the whole stool sample must look and/or feel the same.

   Note: Because we will examine the stool samples with different diagnostic techniques, it is important to obtain a homogenous distribution of the eggs in the stool samples.

2. After each sample of a batch has been homogenization you can proceed with the downstream diagnostic techniques: Duplicate Kato-Katz smears, Mini-FLOTAC and FECPAK^{G2}.
