# Supplementary material for: Comprehensive evaluation of stool-based diagnostic methods and benzimidazole resistance markers to assess drug efficacy and detect the emergence of anthelmintic resistance: A Starworms study protocol
Source: PLoS Negl Trop Dis. 2018 Nov 2;12(11):e0006912. doi: 10.1371/journal.pntd.0006912 (PMC6235403; doi:10.1371/journal.pntd.0006912)
Supplement: S3 Info — (PDF) [file pntd.0006912.s003.pdf]

## Preparation of glycerol-malachite green soaked cellophane clippings.

### 1. Purpose

This SOP describes the preparation of glycerol-malachite green soaked cellophane clippings to be used in the Kato-Katz procedure (SOP 06). The glycerol-malachite green soaked cellophane clippings should be prepared at least 24 h before use.

### 2. Equipment and reagents

- Malachite green (crystals)
- Glycerol 100%
- Cellophane
- Bottled water
- Weighing scale
- Piece of paper
- Marker
- Scalpel or a pair of scissors

### 3. Procedures

#### *3.1. Preparation of a 1% malachite green stock solution*

1. Place a piece of paper on the weighing scale, press 'Tare' and weigh 1.0 gram of malachite green crystals.
2. Add the crystals to 100 ml of bottled water in a sealable bottle and thoroughly mix the suspension.
3. Label as '1% malachite green stock solution + date of preparation'.

#### *3.2. Preparation of a glycerol-malachite green working solution*

1. Add 100 ml of bottled water to a clean bottle.
2. Add 1 ml of 1% malachite green stock solution.
3. Add 100 ml of glycerol (100%) and thoroughly mix the suspension.
4. Label as 'glycerol-malachite green working solution + date of preparation'.

#### *3.3. Preparation of glycerol-malachite green soaked cellophane clippings*

1. Cut cellophane into clippings with a width equal to the width of a microscope slide and a length of approximately 2/3 of a microscope slide.
2. Place the cellophane clippings in an empty recipient, (e.g. a Petri dish or an old glass food jar), and soak the clippings in glycerol-malachite green working solution for at least 24 h.

   Caution: keep the glycerol-malachite green soaked clippings out of direct sunlight.

### 4. References.

This SOP is based on the SOP of Peter Steinmann, Swiss Tropical & Public Health Institute.

# Duplicate Kato-Katz smears

## 1. Purpose

The Kato-Katz thick smear method is the most widely used technique to detect and quantify eggs of soil-transmitted helminths in stool. This SOP describes the procedures to prepare, time and read duplicate Kato-Katz smears.
We will use the **duplicate** Kato-Katz thick smear method, because it has a higher sensitivity compared to a single Kato-Katz smear.
We will record the time needed to prepare duplicate Kato-Katz smears in batches of 10 stool samples. In case a batch contains less than 5 samples, no timing of the preparation is required. Reading of all duplicate Kato-Katz smears will be timed on an individual basis.

## 2. Equipment

- scrap paper or a newspaper
- a 1.5 mm thick template with a hole of 6 mm
- a flat-sided spatula
- microscope slides
- a nylon screen with a mesh size between 60 and 105 μm
- glycerol-malachite green soaked cellophane clippings (see SOP 04 Preparation of glycerol-malachite green soaked clippings)
- a wooden tongue depressor/spatula
- a microscope slide box
- a compound microscope
- multi-counter with at least three counters, one for each soil-transmitted helminth species (*Ascaris*, *Trichuris* & hookworm)
- timer

## 3. Forms

| | |
|---|---|
| RF 01 | Kato-Katz preparation |
| RF 02 | Kato-Katz examination |

## 4. Procedures

### *4.1. Preparation of duplicate Kato-Katz smears of a BATCH OF 10 STOOL SAMPLES*

1. Start the timer
2. Place a small mound (< 1 gram) of stool on the scrap paper or newspaper.
3. Mark the slide as 'Subject ID - A'. For example: ET065 – A.
4. Place the template with the hole on the center of the marked microscope slide.
5. Press a piece of small nylon screen on top of the stool so that part of the stool is sieved through the mesh and accumulates on top.
6. Scrape the sieved stool from the upper surface of the screen using the spatula.
7. Fill the hole of the template on the microscope slide completely with stool from the spatula and remove any excess stool with the spatula.
8. Carefully remove the template by lifting it vertically. Avoid horizontal movements. You will notice that a cylinder of stool is left on the slide.
9. Place a pre-soaked cellophane clipping on top of the stool aliquot on the microscope slide.
10. Caution: remove excessive glycerol-malachite green solution using tissue paper before placing the cellophane clipping on the aliquot.
11. Take a second, clean, microscope slide and place it on top of the cellophane. Press the top microscope down so the stool aliquot spreads evenly. Avoid lifting, wrinkling or moving the cellophane when spreading the smear.
12. Caution: support the slide from below to avoid cracking/breaking.
13. Place the slide in the microscope slide box.
    Caution: keep slides out of direct sunlight!
14. Mark a second microscope slide as 'subject ID - B'.
15. Place the same template in the middle of the second marked slide and make a second smear as described above in steps 6 – 11.
16. Discard the scrap paper or newspaper with the stool sample.
17. Repeat steps 2 – 16 to prepare the A and B smear of the remaining samples of the batch (so a total of 20 smears per batch of 10 stool samples).
18. Stop the timer.
19. Fill in the Record Form Kato-Katz preparation (**RF 01**).
20. Examine slides 'A' and 'B' within 30-60 minutes at ambient temperature. By then it should be possible to see the print of your scrap paper/newspaper through the clarified smear.
    Note: It is important that slides are read within the timeframe of 30-60 minutes after preparation! If slides are read at a later time point, hookworm eggs start to disappear and egg counts will not be correct.

### *4.2. Reading of the INDIVIDUAL Kato-Katz smears*

1. Start the timer
2. Take a smear, either the A or the B smear, examine the smear systematically and count the number of egg for each soil-transmitted helminth species (*Ascaris*, *Trichuris* & hookworm) separately using the multi-counter.
   Note: You should not read smears A and B of the same subject.
   Note: Counting eggs of other parasite species is not required.
3. After reading a single smear (smear A or smear B), stop the timer.
4. Record the number of eggs in Record Form Kato-Katz examination (**RF 02**).
   Record the time it took you to read the smear on the Record Form Kato-Katz examination (**RF 02**).
   **Note:** Avoid reading two smears of the same individual. Leave the second smear to a colleague.
5. After finishing the smear, store the smear in a slide box.
