# Supplementary material for: Comprehensive evaluation of stool-based diagnostic methods and benzimidazole resistance markers to assess drug efficacy and detect the emergence of anthelmintic resistance: A Starworms study protocol
Source: PLoS Negl Trop Dis. 2018 Nov 2;12(11):e0006912. doi: 10.1371/journal.pntd.0006912 (PMC6235403; doi:10.1371/journal.pntd.0006912)
Supplement: S4 Info — (PDF) [file pntd.0006912.s004.pdf]

Preparation of flotation solution

1. Purpose

This SOP describes the preparation of a saturated saline solution that is used as a flotation solution for Mini-FLOTAC (SOP 07) and FECPAK^{G2} (SOP 08).
In the starworms study, 138 ml of flotation solution will be needed per stool sample (38 ml for Mini-FLOTAC and 100 ml for FECPAK^{G2}). Therefore, it is crucial to prepare sufficient flotation solution, for example in batches of 5 liters. The flotation solution should be made at least 24 hours in advance, and hence it should be prepared well in advance.

2. Equipment and reagents

- NaCl (Natrium Chloride, salt)
- Tap water
- Bottle, cooking pot of 5 liter that can be heated
- Warm water bath or heating plate
- Hydrometer
- Thermometer (Optional)

3. Procedure for preparation of a batch of 5 liter

1. Heat 5 liter of tap water to 50-60°C using a warm water bath or a heating plate.
2. Add about 2.5 kg of salt and continue stirring until no more salt dissolves into the solution and excess salt settles on the bottom of the bottle.
3. To ensure that the solution is fully saturated, allow the solution to stand **at least 24 h** at room temperature.

   Note: the temperature of the flotation solution has a major impact on the density of the flotation solution. Make sure the flotation solution is **not kept in the sun**.
4. Check whether crystals are absent: if crystals did not dissolve, continue with step 5. If the salt crystals that settled on the bottom of the bottle have been dissolved, heat the solution (50-60 °C), add salt until no more salt dissolves into the solution and excess salt settles on the bottom of the bottle. Let the solution stand for **at least 24 h** and repeat step 4.
5. Check the specific gravity with a hydrometer. The specific gravity of the saturated solution should be **at least 1.20**.

4. References

Cringoli *et al*. 2010. FLOTAC: new multivalent techniques for qualitative and quantitative copromicroscopic diagnosis of parasites in animals and humans. Nat Protoc. 2010 Mar;5(3):503-15.

Mini-FLOTAC

1. Purpose

The Mini-FLOTAC technique is recently developed to detect and quantify eggs of soil-transmitted helminths in stool. This technique is included in the starworms project as it has proven to be more sensitive compared to duplicate Kato-Katz smears.

This SOP describes the procedures to prepare and read the Mini-FLOTACs.

We will record the time needed to prepare Mini-FLOTACs for batches of 10 stool samples. In case a batch contains less than 5 samples, no timing of the preparation is required.
Reading of the Mini-FLOTACs will be timed on an individual basis.

2. Equipment for analyzing 1 batch of 10 stool samples

- 10 Fill-FLOTACs

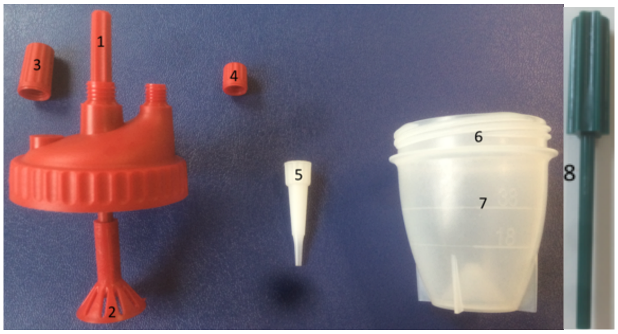

(1: applicator; 2: stool collector; 3: cap of applicator; 4: cap of hole where pipet tips is placed on; 5: pipet tip; 6: stool container; 7: 38 ml line; 8: stick)

- 10 Mini-FLOTAC apparatuses

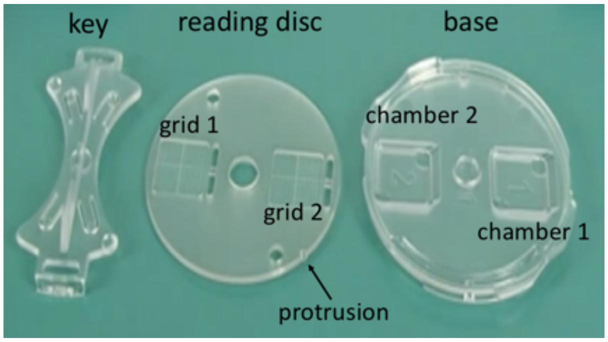

- two timers

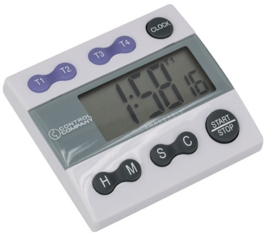

- gloves
- paper tape
- 10 wooden tongue depressors
- a compound microscope
- flotation solution (with a specific density ≥-of at least 1.20; see SOP 03 Preparation of flotation solution).
- a multi-counter with at least three counters, one for each soil-transmitted helminth species (*Ascaris*, *Trichuris* and hookworm)
- a 1 cm thick rectangular item or book to hold the Mini-FLOTAC in an oblique position (optional)

3. Forms

RF 04	Mini-FLOTAC preparation
RF 05	Mini-FLOTAC examination

4. Procedures

4.1. Assembling the Mini-FLOTACs

1. Put on a pair of gloves.
2. The Mini-FLOTAC apparatus consists of three parts: the key, the reading disc with two square grids and the base with two numbered square flotation chambers.

3. We will first assemble the base and the disc: at the top of the base you find a groove; at the bottom of the reading disc you see a protrusion. Put the reading disc on the base, so that the protrusion fits into the groove. You should now be able to turn the disc.

<table>
<tr><td>Mini-FLOTAC apparatus
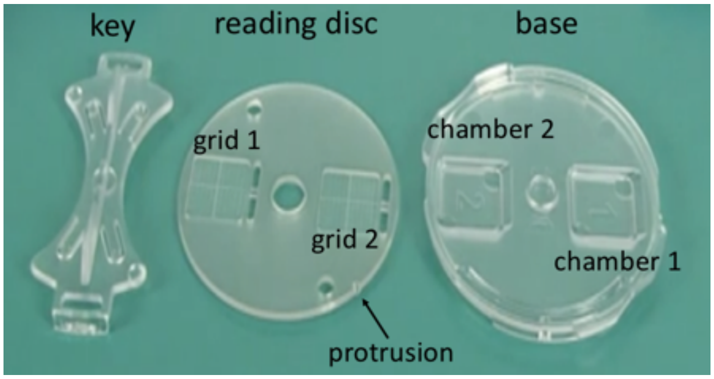
</td><td>Protrusion and groove of device.
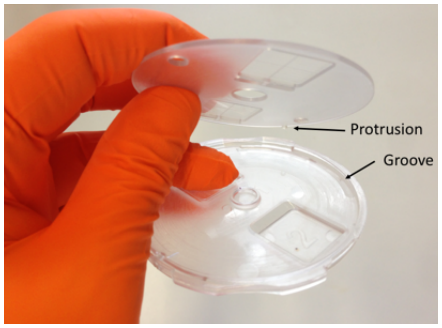
</td></tr>
</table>

4. In order to add the key, you first need to turn the disc in such a way that the grids of the disc are perpendicular to the chambers of the base. Notice the holes next to the chambers.

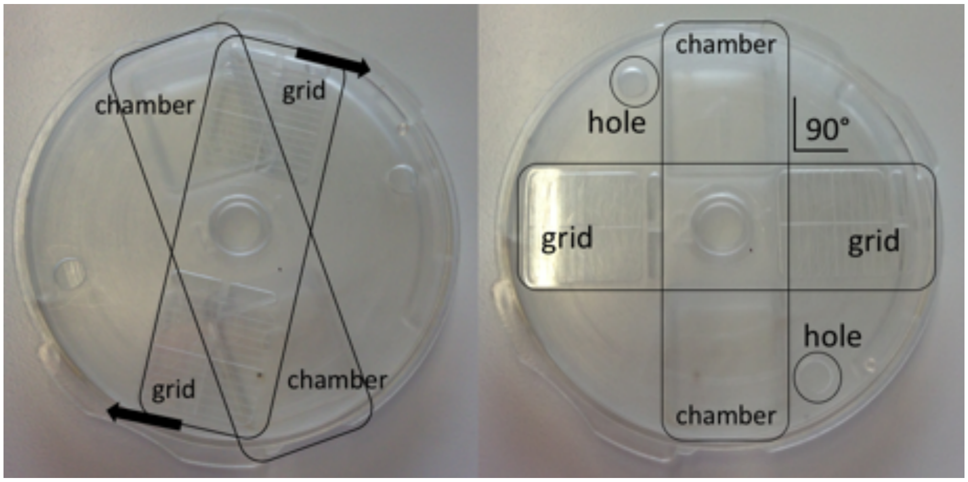

5. Place the matching protrusions at the bottom of the key in the holes next to the chambers.

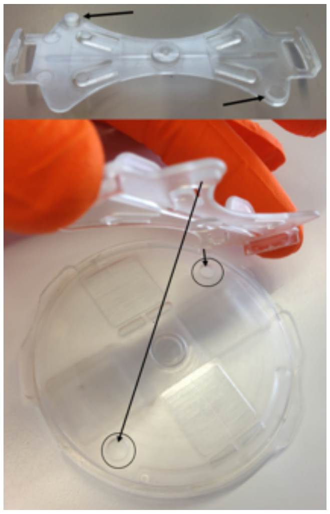

6. Use the key to turn the disc so that the grids are situated above the chambers. At this stage, the key can no longer be disconnected from the disc.

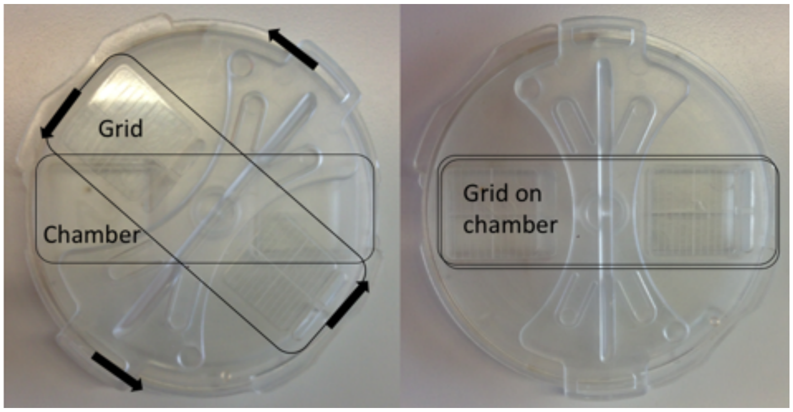

4.2. Preparing a BATCH of 10 STOOL SAMPLES

1. <u>Start the timer</u>.

2. Per batch of 10 stool samples, take 10 clean Fill-FLOTACs, paste a piece of paper tape on the containers and write the sample ID on it.

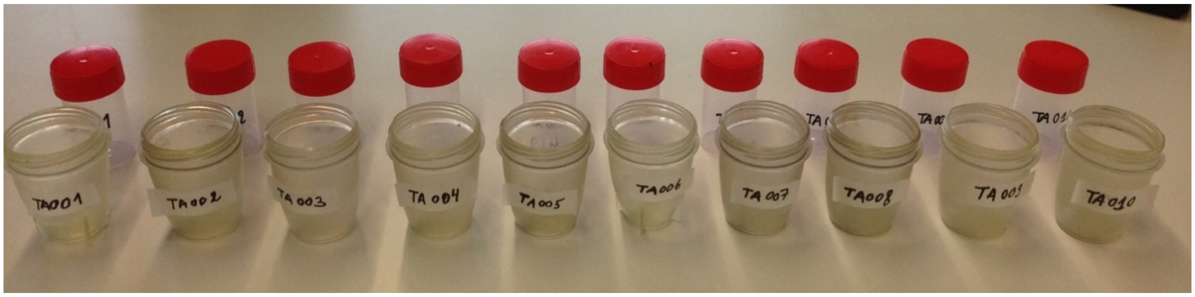

3. Open the Fill-FLOTACs and add flotation solution up to the 38 ml line (specific density ≥ 1.20).

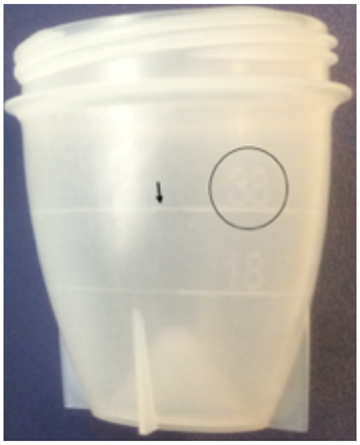

4. Fill the conical collectors of the Fill-FLOTACs using a wooden tongue depressor and level the surface.

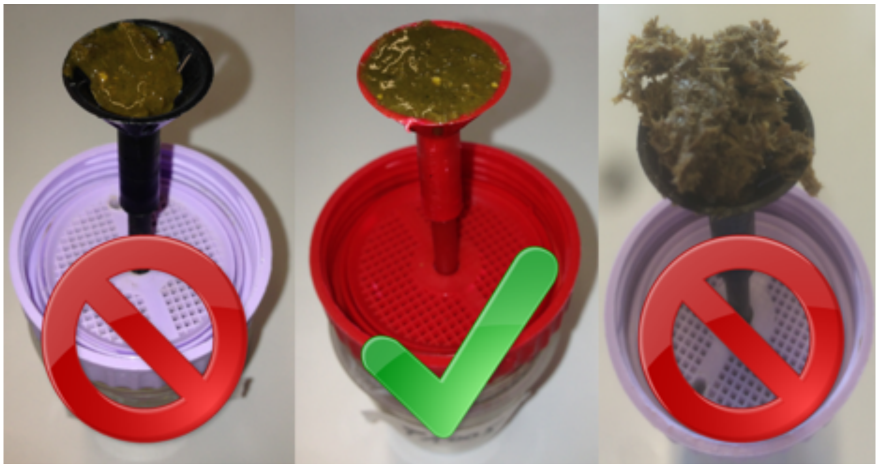

Left: not full enough, middle: enough, right: too full.

5. Close all Fill-FLOTACs and remove the cap of the applicators.

6. Thoroughly mix the suspension by moving the applicator up and down **WHILE** rotating the applicator, until the sample is completely homogenized. Perform this step for all samples of the batch.

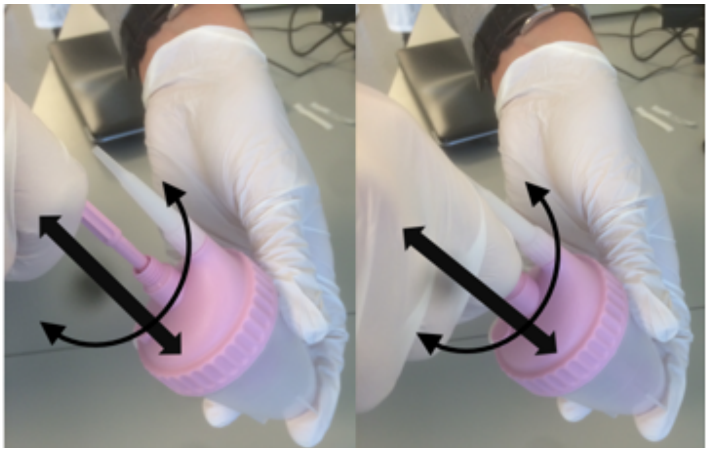

7. In order to fill the Mini-FLOTACs, it is recommended to tilt the Mini-FLOTAC using a +/- 1 cm thick rectangular item. Make sure that the slits of the reading disc are on top of the chambers.

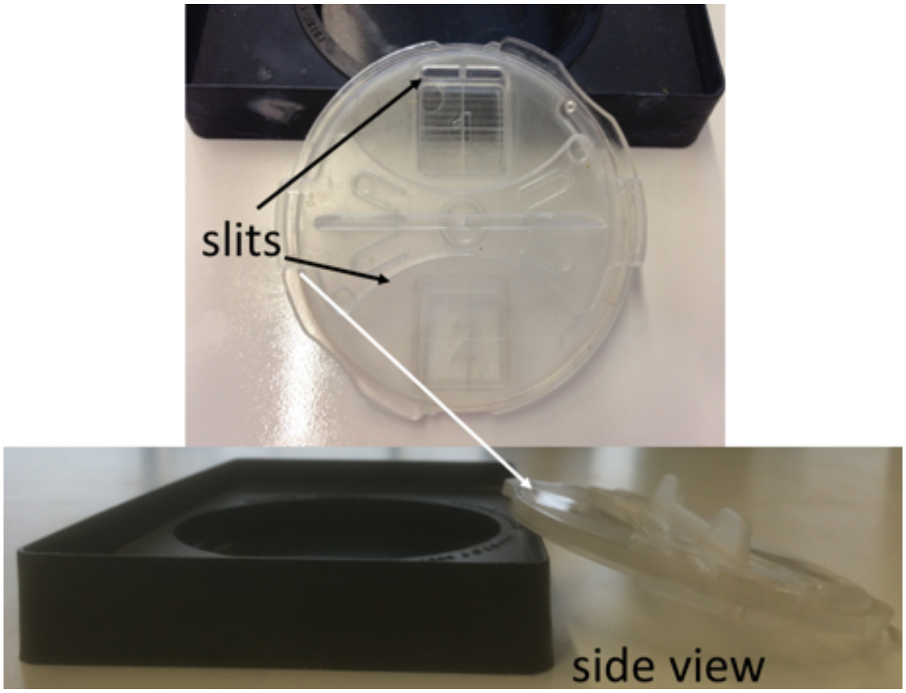

8. Take the Fill-FLOTAC, remove the cap of the tip opening and put a pipette tip on the opening.

9. Homogenize the suspension by inverting the Fill-FLOTAC **5** times.

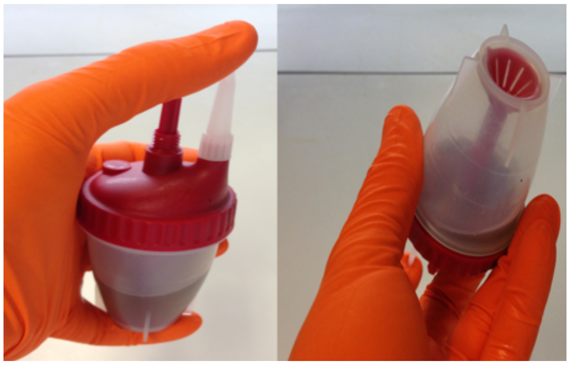

10. Fill the first chamber by squeezing the suspension from the Fill-FLOTAC in the slits of the Mini-FLOTAC. Make sure you keep the tip on the bottom of the device (if not, solution will flow out of Fill-FLOTAC through the opening of the homogenization rod).

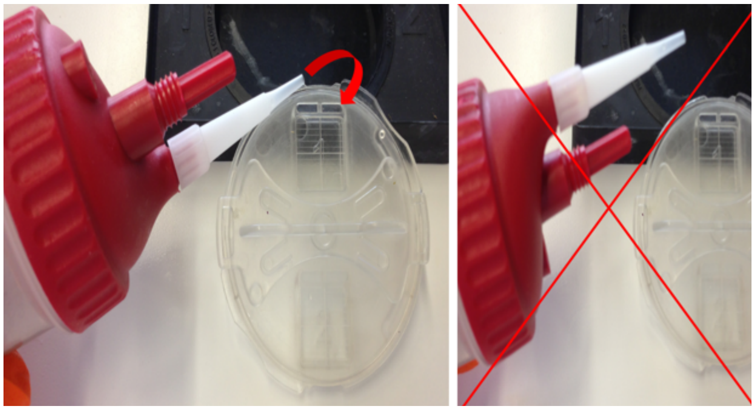

<u>Caution</u>: a blurry vision might appear when reading the grids because of spilt sample fluid on or below the Mini-FLOTAC chambers.

<u>Caution</u>: keep Mini-FLOTACs next to the corresponding labeled Fill-FLOTACs and make sure the Mini-FLOTACs and Fill-FLOTACs are not mixed!

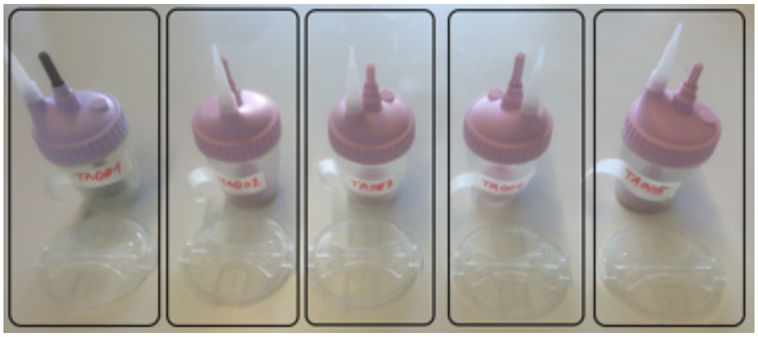

11. Invert the Fill-FLOTAC **5** times and fill the second chamber.

12. Repeat steps 7 – 11 to fill all Mini-FLOTACs of the batch.

13. After filling the 10th (or last) Mini-FLOTAC of the batch, **stop the timer**.

14. Complete the Record Form Mini-FLOTAC preparation **(RF 04)**.

15. Set a timer for 10 minutes after the last Mini-FLOTAC has been filled. After this incubation period of 10 minutes at room temperature, use the keys to turn the reading discs 90°. The grids are now perpendicular to the chambers. You can remove the key but keep the base and the disc together.

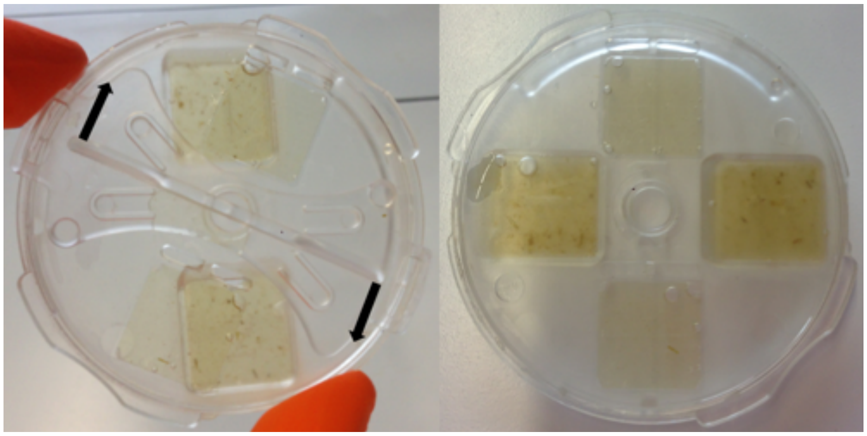

Caution: Do not press the base and reading disc or key. To turn the key, keep the base in the palm of your hand or keep the base between thumb and finger in the two notches.

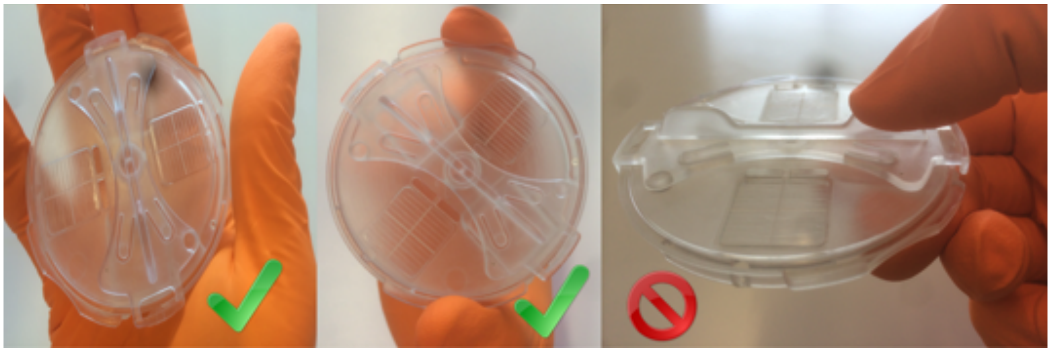

4.3 *Reading of the INDIVIDUAL Mini-FLOTACs*

1. Start a timer.
2. Place the base and reading disc in the microscope holder with the chambers sliding into the gap in the middle of the microscope holder.

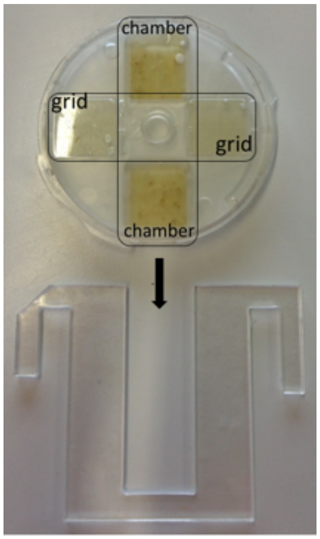

3. Focus on the air bubbles. Systematically screen the two grids of a Mini-FLOTAC for the presence of helminth eggs (*Ascaris*, *Trichuris*, hookworm) using the 10X oculars and count the eggs, seperately for each of the three soil-transmitted helminths, using the multi-counter
4. Stop the timer after reading the two chambers of the Mini-FLOTAC and complete the Record Form Mini-FLOTAC examination (**RF 05**).

4.4 *Cleaning of the Mini-FLOTACs and Fill-FLOTACs*

1. To clean the Mini-FLOTAC, you will need to disassemble the Mini-FLOTACs: turn the disc again until the grids are aligned to the chambers. Use the key to remove the disc.

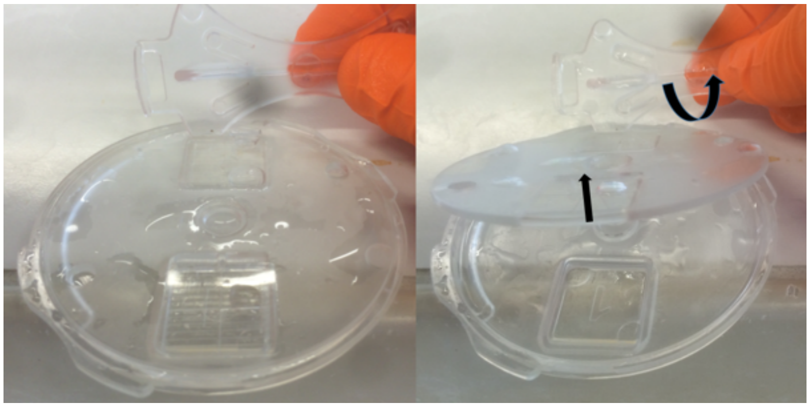

2. Disassemble the filter part of the Fill-FLOTACs by pushing the filter down using the green stick.

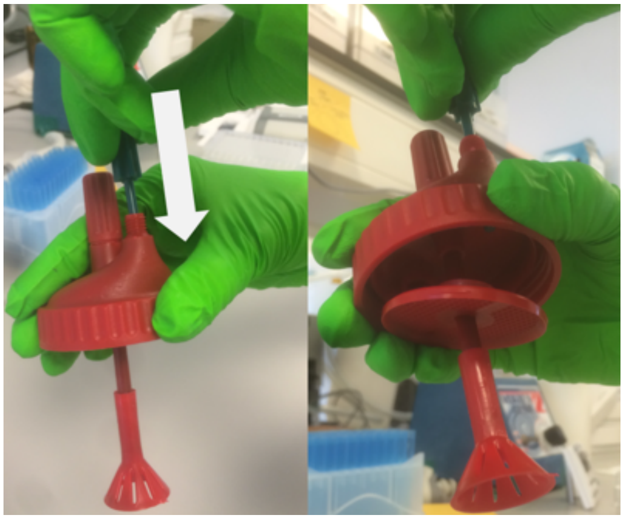

3. Thoroughly wash the Mini-FLOTACs and Fill-FLOTACs for re-usage or let them soak in water.

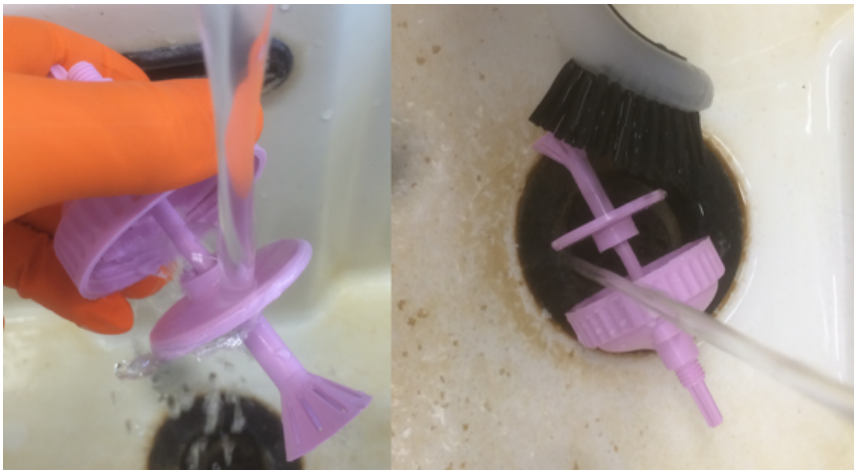

Troubleshooting

Problem: **Large air bubbles are present in one or two chambers after filling the chambers.**

Solution: Tick with fingers against the Mini-FLOTAC and refill. Always homogenize before filling. If this does not help, take a different Mini-FLOTAC apparatus and restart the fill procedure.

Problem: **Large air bubbles are present in one or two chambers after turning the key, before reading the Mini-FLOTACs.**

Solution: These air bubbles are difficult to remove. Note the size of the air bubbles in the comments field of the Record Form Mini-FLOTAC Examination (RF 05).

Air bubble with size < 1/16 of a chamber.

Air bubble with size +/- 1/16 of a chamber.

Air bubble with size +/- ¼ of a chamber.

For bigger air bubbles: fill a new Mini-FLOTAC device.

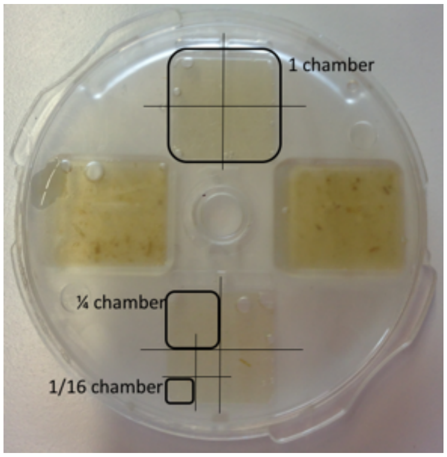

Problem: **Blurry image upon microscopic examination.**

Solution: The image might be blurry upon microscopic examination because of saturated salt solution on or below the reading chambers.

This can be avoided when filling the Mini-FLOTAC (see steps 4.2.7-4.2.11).

Wipe the solution with wet tissue paper and start again with reading of the Mini-FLOTAC.
