# Supplementary material for: Comprehensive evaluation of stool-based diagnostic methods and benzimidazole resistance markers to assess drug efficacy and detect the emergence of anthelmintic resistance: A Starworms study protocol
Source: PLoS Negl Trop Dis. 2018 Nov 2;12(11):e0006912. doi: 10.1371/journal.pntd.0006912 (PMC6235403; doi:10.1371/journal.pntd.0006912)
Supplement: S6 Info — (PDF) [file pntd.0006912.s006.pdf]

Quality Control of Kato-Katz and Mini-FLOTAC

1. Purpose

This SOP describes the procedures for the correct implementation of a quality control on the egg counts using Kato-Katz and Mini-FLOTAC. Using predefined random numbers, 10% of the Kato-Katz smears and Mini-FLOTACs are selected and have to be re-read by a second independent examiner. This SOP defines the quality control criteria and what to do in case these criteria are not met.

2. Equipment

- Compound microscope
- Multi-counter

3. Forms

RF 02	Kato-Katz examination
RF 03	Kato-Katz re-examination
RF 05	Mini-FLOTAC examination
RF 06	Mini-FLOTAC re-examination

4. Procedures

Table 1 contains the random subject ID numbers of which the Kato-Katz smears AND the Mini-FLOTACs need to be read by a second independent examiner during the baseline survey.

Before follow-up starts, a new table will be generated using the IDs of those subjects who were positive for any of the three soil-transmitted helminths at baseline (*Ascaris*, *Trichuris* & hookworm). This table will be generated by the Ghent team once all baseline data are available.

Table 1: Random subject ID numbers to be re-examined by Kato-Katz and Mini-FLOTAC.

13	228	435	615	811
19	259	437	634	819
34	270	441	653	823
38	285	446	675	
54	292	449	686	
59	317	456	687	
64	326	468	691	
76	337	472	695	
79	340	473	735	
80	345	480	743	
94	349	481	749	

95	353	482	753	
107	357	485	754	
134	359	525	757	
157	363	569	758	
168	376	576	759	
182	389	583	761	
189	397	595	767	
195	404	598	783	
223	422	611	790	

4.1. Quality control of Kato-Katz smear egg counts

1. Select the Kato-Katz smears according to Table 1. In case of follow-up samples, the numbers will be generated later on.
2. Examine the selected Kato-Katz smears 'A' and 'B' systematically and count the number of eggs of each species (*Ascaris*, *Trichuris*, hookworm) using the multi-counter.

   Caution: It is important that slides are read within the timeframe of 30-60 minutes after preparation! If slides are read at a later time point, there is over-"clearing" of hookworm eggs and egg counts will not be correct for this parasite.

   Note: Counting eggs of other species is not required.

   Note: Timing of this procedure is not required.
3. Record the number of eggs in the Record Form Kato-Katz – re-Examination (RF 03), use the column '**Second exam.**'.
4. Copy eggs counts from the first examiner, recorded in Record Form Kato-Katz examination (RF 01) in the column 'First exam.'
5. Compare your eggs counts with those of the first examiner and check if quality criteria are met:
   - No false negatives/positives;
   - difference in egg counts is ≤10 eggs when the total number of eggs counted is ≤100 eggs (not eggs per gram of stool, but number of eggs counted under the microscope)
   - difference in egg counts is ≤20% when more than 100 eggs are counted.
6. If the smear does not meet the quality criteria, thick the box 'No' and resolve the discrepancy with the first examiner as described in Table 2. If the quality criteria are met, thick 'yes' and continue.

Table 2: Quality criteria for eggs counts (Kato-Katz and Mini-FLOTAC).

Quality criterion	What to do if criterion is not met
No false negatives/positives	Examiner of the positive slide has to show the detected eggs to the examiner who did not detect the egg. After discussion, a decision has to be made on positivity/negativity of the slide.<br>**On the Record Form Kato-Katz re-examination (<u>RF 03</u>)**: strikethrough the incorrect count<br>**On the Record Form Kato-Katz examination (<u>RF 02</u>):** strikethrough and write the correct count if the count was incorrect.
Difference in egg counts: ≥10 eggs difference when the total number of eggs counted by the **first** examiner ≤100 eggs<br>(not eggs per gram of stool, but number of eggs counted under the microscope)	A third examiner should count the eggs to confirm the egg counts of examiner 1 or 2.<br>**On the Record Form Kato-Katz re-examination (<u>RF 03</u>):** strikethrough the incorrect count.<br>**On the Record Form Kato-Katz examination (<u>RF 02</u>)**: strikethrough and write the correct count if the count was incorrect.
Difference in egg counts: ≥20% when more than 100 eggs are counted by the **first** examiner	A third examiner should count the eggs to confirm the eggs counts of examiner 1 or 2.<br>**On the Record Form Kato-Katz re-examination (<u>RF 03</u>)**: strikethrough the incorrect count.<br>**On the Record Form Kato-Katz examination (<u>RF 02</u>):** strikethrough and write the correct count if the count was incorrect.

7. Examples:

<u>Example 1</u>: examiner 1 counts 10 *Trichuris* eggs, examiner 2 counts 30 *Trichuris* eggs. The total number of counted eggs by the first examiner is 10 (≤100). The difference in egg counts between the two examiners is 20 (30-10). This difference is bigger than 10, so the quality criteria are not met.

A third examiner is invited to count the *Trichuris* eggs, and he/she counts 32 *Trichuris* eggs. This means there is no difference in eggs counts (according to the definitions stated in Table 2) between examiner 3 and examiner 2, confirming the eggs counts of examiner 2. In the re-examination form, the incorrect count of examiner 1 is corrected by striking through the incorrect count. In the examination form, the incorrect count is also crossed out, and the correct count is written down (also see SOP 01 General information on how to complete form).

Example 2: examiner 1 detected a *Trichuris* egg, examiner 2 did not detect any *Trichuris* egg. There is a false positive or a false negative result, so the quality criteria 1 is not met. Examiner 2 invites examiner 1 to show him or her the *Trichuris* egg. Examiner 1 shows a presumed *Trichuris* egg, but examiner 2 identifies this as an artefact. Examiner 1 agrees and the re-examination and examination forms are adjusted accordingly.

4.2. Quality control of Mini-FLOTAC egg counts

1. Select the Mini-FLOTACs according to the randomization scheme (Table 1). In case of follow-up samples, select the Mini-FLOTACs according to a newly designed table that will be communicated later on.
2. Read the selected Mini-FLOTAC devices as described in SOP 07.
3. Record the egg counts on the Record Form Mini-FLOTAC – **re-**Examination (RF 06). Use the column '**Second exam.**'.
4. Fill in eggs counts from the first examiner, recorded in the Record Form Mini-FLOTAC examination (RF 05) in the column 'First exam.'
5. Compare your eggs counts with those of the first examiner and check if quality criteria are met (Table 2).
6. If the egg counts do not meet the quality criteria, thick the box 'No' and resolve the discrepancy with the first examiner as described in Table 2 and exemplified above. If the quality criteria are met, thick 'yes' and continue.
