# Supplementary material for: Comprehensive evaluation of stool-based diagnostic methods and benzimidazole resistance markers to assess drug efficacy and detect the emergence of anthelmintic resistance: A Starworms study protocol
Source: PLoS Negl Trop Dis. 2018 Nov 2;12(11):e0006912. doi: 10.1371/journal.pntd.0006912 (PMC6235403; doi:10.1371/journal.pntd.0006912)
Supplement: S7 Info — (PDF) [file pntd.0006912.s007.pdf]

Preservation of stool samples

1. Purpose

This SOP describes the procedures for the preservation of stool samples after the performance of the different diagnostic techniques used in this study.

During baseline evaluation, only stool samples found to contain at least 13 eggs on duplicate Kato-Katz or at least 15 eggs on Mini-FLOTAC for at least one of the three soil-transmitted helminths (*Ascaris*, *Trichuris* & hookworm) will be preserved

During follow-up evaluation, all stool samples will be preserved from all subjects.

The stool samples will be preserved in duplicate, using two different preservation solutions, i.e. 100% ethanol and 5% Potassium Dichromate.

These stool samples will be shipped to Ghent University, Belgium for further molecular analysis.

2. Equipment

- 100% ethanol
- 5% Potassium Dichromate (see SOP 15 'Preparation of 5% Potassium Dichromate').
- a plastic beaker of at least 150 ml
- 2 ml Eppendorfs
- a scale
- a pipette of 1 ml
- a small wooden applicator or thin spatula
- parafilm

3. Forms

LF 02	Stool preservation
RF 02	Kato-Katz examination
RF 05	Mini-FLOTAC examination

4. Procedures

1. Check the Record Forms Kato-Katz examination and Mini-FLOTAC examination (RF 02 and RF 05) to identify the stool samples that need to be preserved (see last column of both Record Forms: 'Selected for stool preservation?').
2. For every stool sample to be preserved, a total of **four** 2 ml Eppendorf tubes needs to be labeled: two with the 'subject identifier + BL (for baseline samples) or FU (for follow-up samples)' + '**E**' (for **e**thanol preservation), and two with the 'subject identifier + BL (for baseline samples) or FU (for follow-up samples)' + '**P**' (for 5% **P**otassium Dichromate preservation).

   For example, the following tubes are required for a baseline stool sample from Subject ID 'TA002':

TA002-BL-E	TA002-BL-P
TA002-BL-E	TA002-BL-P

3. Make 4 aliquots of **exactly 0.5 gram**: place an Eppendorf on the scale, press the tare key and add exactly 0.5 gram of stool to the 2 ml Eppendorf using a wooden applicator or a small spatula. Repeat for the three other Eppendorfs.
4. To avoid contamination of the stock of the preservation solutions pure ethanol or 5% Potassium Dichromate, it is recommended to transfer a small volume of each into two plastic beakers, one for ethanol and one for 5% Potassium Dichromate.
5. With a pipette, add 1 ml of pure ethanol to each of the two tubes labelled **'E'** and mix the suspension by shaking the closed tube.

   Note: If insufficient stool was provided, then preserve at least one stool sample with pure ethanol and the other one in 5% Potassium Dichromate.
6. With a pipette, add 1 ml of 5% Potassium Dichromate solution to each of the two tubes labelled **'P'** and mix the suspension by shaking the closed tube.
7. Put parafilm around the caps to avoid the evaporation of the ethanol during storage or shipment.
8. Store at room temperature or at 4 °C in boxes until shipment. Place the pure ethanol preserved samples (labeled as 'E') in a box labeled 'stool ethanol preservation' and the stool samples preserved in 5% Potassium Dichromate (labeled as 'P') in a box labeled 'stool Potassium Dichromate preservation'.
9. Complete the Log Form Stool preservation (**LF 02**).
10. This procedure should be repeated daily for all selected stool samples.
