# Supplementary material for: Comprehensive evaluation of stool-based diagnostic methods and benzimidazole resistance markers to assess drug efficacy and detect the emergence of anthelmintic resistance: A Starworms study protocol
Source: PLoS Negl Trop Dis. 2018 Nov 2;12(11):e0006912. doi: 10.1371/journal.pntd.0006912 (PMC6235403; doi:10.1371/journal.pntd.0006912)
Supplement: S8 Info — (PDF) [file pntd.0006912.s008.pdf]

DNA extraction and purification of ethanol preserved stool samples

1. Purpose

This SOP describes the procedures to extract and purify DNA from individual and pooled stool samples preserved in ethanol (96%-100%). The procedures for taking subsamples and pooling of samples are described in SOP 18 ‘Pooling of ethanol preserved stool samples’.

2. Equipment and reagents

- 1000 μl pipet tips with filter
- 1000 μl pipet
- Pair of scissors
- Phosphate buffered saline (PBS)
- ATL buffer (Qiagen)
- Proteinase K (PK) (Qiagen)
- PVPP (Polyvinylpyrrolidone; Fluka 77627; Sigma)
- Ceramic beads, 1.4 mm (MoBio, Qiagen, reference: 13133-325) (or MagNA Lyser Green Beads Tubes (Roche, 03358941001)).
- 2 ml tubes Sarstedt (reference: 72.694.005)
- Eppendorf tubes
- 50 ml Falcon tubes
- Holder for 2 ml Sarstedt/Eppendorf tubes
- Centrifuge
- TissueLyser (Roche)
- Vortex
- - 20 °C or – 70 °C freezer
- Shaking heat block or warm water bath
- QIAsymphony automated extraction and purification platform (Qiagen)

3. Forms

RF 10	Pooling of ethanol preserved stool samples
RF 11	DNA extraction of ethanol preserved stool samples

4. Procedures

4.1. Organization of samples in batches of 24 and preparation of sample sheets and labels

1. We will work in batches of 24 (the capacity of the centrifuge). Each batch should contain 23 stool samples (individual ones or pooled ones) and one sample of distilled water as negative control for the DNA extraction.
2. Open the RF 11, copy the first sheet (DD-MMM-2017) and save the new sheet (in the same excel file) as the date of today. Fill in extraction date and name.
3. Fill in all samples to be extracted today: fill in Site (BR, Brazil; ET, Ethiopia; LA, Laos; TA, Tanzania), Participant or Pool number, Visit (BL, baseline; FU, follow-up). This will autogenerate the 'Sample ID'. Choose 'XXX' for individual samples, and P10, P20 or P60 for pooled samples of 10, 20 or 60, respectively. Make sure every 24th sample is a negative control (every 24th position in RF 11 is prepopulated as 'negative control').
4. Fill in the number of samples in the top left corner.
5. Print QR labels using the 'Sample ID' according to SOP 20 'Printing of QR Labels'.
6. Print the sample sheet.
7. Organize the samples to be extracted into batches of 24 in Eppendorf tube holders.
8. Preheat the warm water bath at 55 °C.

4.2. Preparation of buffers and reagents

1. Check if the buffers and reagents needed for today's extractions are sufficient enough. If yes, complete the lot numbers in RF 11; if not, continue with the steps below.
2. Preparation of PBS: add one tablet of PBS into a sterile 200 ml bottle and add 200 ml of distilled water. Divide into 4 Falcon tubes of 50 ml and label these as 'PBS + DD/MMM/YYYY'. One Falcon tube containing 50 ml of PBS is sufficient for the extraction of one batch of 24 samples (1 ml in step 'In advance, step 12' and 1 ml in 'Procedures, step x). Complete the lot number (date) in RF 11.
3. Preparation of a 2% PVPP solution (w/v): weigh 1 gram of PVPP in a 50 ml Falcon tube and add 50 ml of PBS. Gently mix until a homogenous suspension is obtained, divide into two aliquots of 25 ml in 50 ml Falcon tubes and label as '2% PVPP + DD/MM/YYYY'. One Falcon tube containing 25 ml of 2% PVPP solution is sufficient for the extraction of two batches of 24 samples. Complete the lot number (date) in RF 11.
4. Preparation of ATL-PK buffer: pipet 5 ml of Proteinase K into a 50 ml Falcon tube. Add ATL buffer until the 50 ml line and gently mix the solution. Divide in two aliquots of 25 ml in 50 ml Falcon tubes. Label as 'ATL/PK + DD/MM/YYYY'. One Falcon tube containing 25 ml of ATL-PK solution is sufficient for the extraction of two batches of 24 samples. Complete the lot number (date) in RF 11.

4.3. Washing steps

1. Centrifuge for 1 minute 30 seconds at 10,000 rpm (9,500 g).
2. Remove the supernatant using filter tips.

4.4. Freezing and bead beating

1. Add 500 μl of 2% PVPP using filter tips, close the tube and resuspend the pellet gently by shaking. Remark: PVPP does not stay dissolved, so you have to stir it regularly while using it.
2. Freeze the sample for 30 minutes at – 70°C or overnight at – 20 °C.
3. Thaw the sample and place in the TissueLyser for 30 seconds at a frequency of 3,000 s^{-1}.
4. Make a short spin.
5. Add 500 μl ATL-PK solution using filter tips and gently resolve by shaking.
6. Incubate for 2 hours (or overnight) at 55 °C in a shaking heat block or warm water bath.
7. Centrifuge at 14,000 rpm (18,000 g) for 1 minute.

4.5. Transfer and labeling

1. Transfer supernatant in a new 2 ml Sarstedt tube using filter tips.
2. Label using the printed QR labels.
3. Proceed with the DNA/RNA isolation using the QIAsymphony DSP Virus/Pathogen Midi Kit.

5. References

Kaisar MMM, Brienen EAT, Djuardi Y, Sartono E, Yazdanbakhsh M, Verweij JJ, Supali T, Van Lieshout L. 2017. Improved diagnosis of *Trichuris trichiura* by using a bead-beating procedure on ethanol preserved stool samples prior to DNA isolation and the performance of multiplex real-time PCR for intestinal parasites. Parasitology 144(7):965-974.

Mwangi MN, Roth JM, Smit MR, Trijsburg L, Mwangi AM, Demir AY, Wielders JP, Mens PF, Verweij JJ, Cox SE, Prentice AM, Brouwer ID, Savelkoul HF, Andang'o PE, Verhoef H. 2015. Effect of daily antenatal iron supplementation on *Plasmodium* infection in Kenyan women: a randomized clinical trial. JAMA 8;314(10):1009-20.
