# Supplementary material for: Comprehensive evaluation of stool-based diagnostic methods and benzimidazole resistance markers to assess drug efficacy and detect the emergence of anthelmintic resistance: A Starworms study protocol
Source: PLoS Negl Trop Dis. 2018 Nov 2;12(11):e0006912. doi: 10.1371/journal.pntd.0006912 (PMC6235403; doi:10.1371/journal.pntd.0006912)
Supplement: S9 Info — (PDF) [file pntd.0006912.s009.pdf]

Absolute quantification of soil-transmitted helminths (STH) by means of quantitative Polymerase Chain Reaction (qPCR).

1. Purpose

This SOP describes the procedures for the absolute quantification of the soil-transmitted helminths (STHs, *Ascaris lumbricoides*, *Trichuris trichiura*, *Necator americanus*, *Ancylostoma duodenale* and *Strongyloides stercoralis*) and *Schistosoma spp.* in DNA extracts obtained from stool using quantitative PCR (qPCR).

2. Equipment and reagents

- Primers and probes (See Table 1).
- Qiagen Quantitect master mix (Qiagen)
- Bovine Serum Albumin (BSA) (Sigma)
- Molecular water
- Standard dilution series for *Ascaris*, *Trichuris* and *Necator* (see SOP 21).
- Rotorgene qPCR platform and Rotorgene Rotor-Discs
- Filter tips and pipets
- Gloves

3. Procedures

1. The qPCR assays targeting the different STHs are designed in two multiplex assays.
    - Multiplex 1 targets *A. lumbricoides*, *N. americanus*, *A. duodenale.*
    - Multiplex 2 targets *T. trichiura*, *Schistosoma* spp. and *Strongyloides stercoralis*.
2. Prepare the amplification reaction mixtures for multiplex 1 and multiplex 2 as described in Table 2.
3. In every multiplex run, add a negative control (ie. reaction mixture without DNA sample) and a positive control for each target (genomic DNA of each target, see SOP 19).
4. To construct a standard curve for the absolute quantification of *A. lumbricoides*, *T. trichiura* and *N. americanus*, add a standard dilutions series for each species (see SOP 20). This should only be done in the first run, in subsequent runs, these standard curves can be imported. Fill in the concentration of the difference dilutions (expressed as genome equivalents per ml of DNA extract (GE/ml) in the software.
5. Perform the amplification on the RotorGene platform using the following cycling conditions for both multiplex assays: initial denaturation of 15 min at 95°C, followed by 45 cycles of 10 s at 95°C, 15 s at 60°C and 15 s at 72°C.
6. Verify the qPCR efficiency (%), calculated by the software based on the slope of the standard curve, which should be in the range 90%-110%.
7. All results are expressed in GE/ml.

Table 1. Primers and probes.

Species	Primer/probe	Sequence (5'-3')	Target region	Ref
A. lumbricoides[a]	Fwd	GTAATAGCAGTCGGCGGTTTCTT	ITS-1	1
	Rev	GCCCAACATGCCACCTATTC		1
	Probe	Texas Red-TTGGCGGACAATTGCATGCGAT-BHQ2		2
T. trichiura[b]	Fwd	TTGAAACGACTTGCTCATCAACTT	18S–ITS1	3
	Rev	CTGATTCTCCGTTAACCGTTGTC		3
	Probe	Yakima Yellow-CGATGGTACGCTACGTGCTTACCATGG-BHQ1		3
A. duodenale[a]	Fwd	GAATGACAGCAAACTCGTTGTTG	ITS-2	4
	Rev	ATACTAGCCACTGCCGAAACGT		4
	Probe*	Cy5-ATCGTTTACCGACTTTAG- BHQ2		4
N. americanus[a]	Fwd	CTGTTTGTCGAACGGTACTTGC	ITS-2	4
	Rev	ATAACAGCGTGCACATGTTGC		4
	Probe*	FAM-CTGTACTACGCATTGTATAC-BHQ1		4
Schistosoma[b]	Fwd	GGTCTAGATGACTTGATYGAGATGCT	ITS2	5
	Rev	TCCCGAGCGYGTATAATGTCATTA		5
	Probe	FAM-TGGGTTGTGCTCGAGTCGTGGC-BHQ1		5
Strongyloides[b]	Fwd	GAATTCCAAGTAAACGTAAGTCATTAGC	SSU	6
	Rev	TGCCTCTGGATATTGCTCAGTTC		6
	Probe	Texas Red-ACACACCGGCCGTCGCTGC-BHQ2		6
Phocine herpes virus[a,b]	Fwd	GGGCGAATCACAGATTGAATC	gB gene	7
	Rev	GCGGTTCCAAACGTACCAA		7
	Probe	Cy5-TTTTTATGTGTCCGCCACCATCTGGATC-BHQ2		7

*Minor groove binding probes. [a]Part of a multiplex qPCR that detects *A. lumbricoides*, *N. americanus*, and *A. duodenale*. [b]Part of a multiplex qPCR that detects *T. trichiura*, *Schistosoma sp*. and *Strongyloides stercoralis*. ITS-1, internal transcribed spacer 1; ITS-2, internal transcribed spacer 2; BHQ1, black hole quencher 1; BHQ2, black hole quencher 2; gB, glycoprotein B.

Table 2. Reaction mixtures for multiplex 1 and multiplex 2 for a single reaction.

Multiplex 1	Multiplex 2
25 µl Qiagen Quantitect mastermix	
2.5 µgram BSA	
10 µl DNA sample	
200 nM *Ancylostoma* primer Fwd	300 nM *Trichuris* primer Fwd
200 nM *Ancylostoma* primer Rev	300 nM *Trichuris* primer Rev
200 nM *Ancylostoma* primer Probe	100 nM *Trichuris* primer Probe
200 nM *Ascaris* primer Fwd	200 nM *Schistosoma* primer Fwd
200 nM *Ascaris* primer Rev	200 nM *Schistosoma* primer Rev
100 nM *Ascaris* primer Probe	100 nM *Schistosoma* primer Probe
300 nM *Necator* primer Fwd	200 nM *Strongyloides* primer Fwd
300 nM *Necator* primer Rev	200 nM *Strongyloides* primer Rev
300 nM *Necator* primer Probe	100 nM *Strongyloides* primer Probe
100 nM PhHV-1 primer Fwd	100 nM PhHV-1 primer Fwd
100 nM PhHV-1 primer Rev	100 nM PhHV-1 primer Rev
100 nM PhHV-1 primer Probe	100 nM PhHV-1 primer Probe

Table 3. Cycling conditions for multiplex 1 and multiplex 2.

Step		Time	Temperature
Initial denaturation		15 min	95 °C
45 cycles	denaturation	10 s	95 °C
	annealing	15 s	60 °C
	elongation	15 s	72 °C

4. References

1. Liu J et al. 2016. Optimization of quantitative PCR methods for enteropathogen detection. PLoS One. 11:e0158199.
2. Wiria AE et al. 2010. Does treatment of intestinal helminth infections influence malaria? Background and methodology of a longitudinal study of clinical, parasitological and immunological parameters in Nangapanda, Flores, Indonesia (ImmunoSPIN study). BMC Infect. Dis. 10:77-2334-10-77.
3. Liu J et al. 2013. A laboratory-developed TaqMan array card for simultaneous detection of 19 enteropathogens. J. Clin. Microbiol. 51:472-480.
4. Verweij JJ et al. 2007. Simultaneous detection and quantification of *Ancylostoma duodenale*, *Necator americanus*, and *Oesophagostomum bifurcum* in fecal samples using multiplex real-time PCR. Am. J. Trop. Med. Hyg. 77:685-690.
5. Obeng BB et al. 2008. Application of a circulating-cathodic-antigen (CCA) strip test and real-time PCR, in comparison with microscopy, for the detection of *Schistosoma haematobium* in urine samples from ghana. Ann. Trop. Med. Parasitol. 102:625-633.
6. Verweij JJ et al. 2009. Molecular diagnosis of *Strongyloides stercoralis* in faecal samples using real-time PCR. Trans. R. Soc. Trop. Med. Hyg. 103:342-346.
7. Niesters HG. 2002. Clinical virology in real time. Journal of Clinical Virology. 25:S3-12.
