# Supplementary material for: Comprehensive evaluation of stool-based diagnostic methods and benzimidazole resistance markers to assess drug efficacy and detect the emergence of anthelmintic resistance: A Starworms study protocol
Source: PLoS Negl Trop Dis. 2018 Nov 2;12(11):e0006912. doi: 10.1371/journal.pntd.0006912 (PMC6235403; doi:10.1371/journal.pntd.0006912)
Supplement: S10 Info — (PDF) [file pntd.0006912.s010.pdf]

## Subsampling and pooling of ethanol preserved stool samples

### 1. Purpose

In the Starworms study, we will compare individual samples to pooled samples for the molecular detection and quantification of soil-transmitted helminths (STHs) infections and mutations in the β-tubulin gene linked to anthelmintic resistance.
This SOP describes the procedures **(i)** to take subsamples of the individual samples for DNA extraction and **(ii)** to pool stool samples preserved in ethanol (96%-100%). We will pool individual samples into pools of 10, 20 and 60. As one of the goals of the Starworms study is to assess the cost-effectiveness of different microscopic and molecular diagnostic techniques, we will time the procedures for both the subsampling and the pooling.

### 2. Equipment and reagents

- Wooden stick to homogenize the stool suspension
- 1000 μl pipet tips with filter
- 1 ml pipet
- Pair of scissors
- 15 ml Falcon tubes
- 5 ml Eppendorf tubes
- 2 ml Eppendorf tubes
- Timer
- Centrifuge
- Vortex
- Labels

### 3. Forms

| | |
|---|---|
| RF 10 | Record Form Pooling and Subsampling of Ethanol Preserved Stool Samples |
| DEF 01 | Data Entry Form 01 |

## 4. Procedures

*Completing RF 10.*

1. Open the RF 10 and select the sheet of the site (BR, ET, LA or TA) and visit (BL or FU) of the planned pooling and subsampling (See Figure 1 below).

Record Form 10 — Pooling of ethanol preserved samples

| Site | Subject | Visit | Sample ID | n° 10P | sample ID 10P | n° 20P | sample ID 20P | n° 60P | sample ID 60P |
|---|---|---|---|---|---|---|---|---|---|
| TA | 001 | BL | TA001_BL | 1 | TA-10P1-BL | | TA-20P1-BL | | TA-60P1-BL |
| TA | 002 | BL | TA002_BL | | TA-10P1-BL | | TA-20P1-BL | | TA-60P1-BL |
| TA | 003 | BL | TA003_BL | | TA-10P1-BL | | TA-20P1-BL | | TA-60P1-BL |
| TA | 004 | BL | TA004_BL | | TA-10P1-BL | | TA-20P1-BL | | TA-60P1-BL |
| TA | 005 | BL | TA005_BL | | TA-10P1-BL | | TA-20P1-BL | | TA-60P1-BL |
| TA | 006 | BL | TA006_BL | | TA-10P1-BL | | TA-20P1-BL | | TA-60P1-BL |
| TA | 007 | BL | TA007_BL | | TA-10P1-BL | | TA-20P1-BL | | TA-60P1-BL |

TA BL | TA FU | ET BL | ET FU | LA BL | LA FU | BR BL | BR FU | +

**Figure 1.** Record Form 10.

2. Open the 'DEF 01' of the corresponding study site, select and copy the Subject IDs. Paste these Subject IDs in the 'Subject' column of the RF 10 (See Figure 1).
3. The pools of 10, 20 and 60 are defined by a unique prepopulated sample ID in the columns 'sample ID 10P', 'sample ID 20P', and 'sample ID 60P', respectively.
4. Use the Sample IDs of the pools to print labels according to SOP 20 'Printing labels'. Labels for the individual samples do not need to be printed, as these samples will be used for DNA extraction and will not be stored (See SOP 19).
5. Print RF 10 for the samples to be pooled in the lab.

*Subsampling and making pools of 10.*

1. For every ethanol-preserved stool sample, cut the top of a 1 ml filter tip using a pair of scissors, leaving a top with an internal diameter of approximately 2 mm.
   *Without cutting the tip, the thick stool suspension will get stuck in the tip when aspirating the stool suspension.*
2. Start timing.
3. Place the samples to be pooled into a pool of ten in a staggered way, as depicted in Figure 2, box 1.
   **Remark:** *In order not to introduce a systematic subsampling bias: of every other ethanol-preserved sample (ie, the samples of the lower row indicated by the white box 1 in Figure 2), we will first take a subsample for individual DNA extractions (in Eppendorfs indicated in the white box 2 in Figure 2), then a second subsample for the pooling (box 3). Of the other samples (ie, the samples in the upper row in box 1), the first subsample will go into the pool (Eppendorf shown in box 3) and the second subsample into the tubes for individual DNA extractions (box 2). The samples are put in a staggered way as a visually aid in this subsampling strategy.*

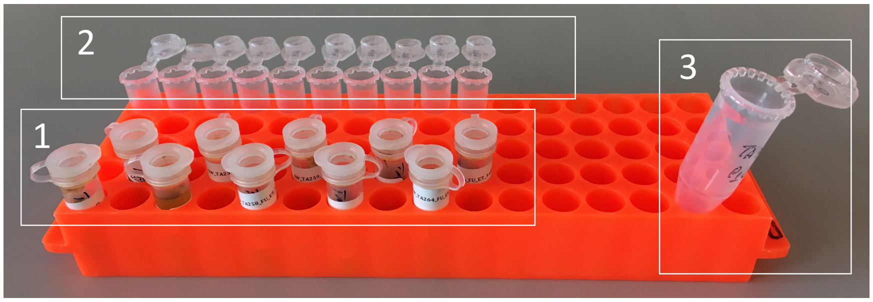


**Figure 2**. Organization of samples to make pools of 10. Box 1, ethanol preserved stool samples; box 2, subsamples for downstream DNA extraction; box 3, pooled sample of 10.

4. Take a 5 ml Eppendorf tube for the pool of 10 (box 3 in Figure 2 above) and label with the pool ID, take ten 2 ml Eppendorf tubes for DNA extraction (box 2 in Figure 2 above) and label with the individual sample ID.
5. Check if the ethanol-stool samples are well suspended. If not, take a wooden stick and gently resuspend the stool in the ethanol until a homogenous suspension is obtained.
6. For the stool samples of the lower row: first aspirate 250 μl of the ethanol-stool suspension into a corresponding Eppendorf tube for individual DNA extraction, then aspirate 250 μl of the ethanol-stool suspension into a 5 ml Eppendorf tube (pooled sample).
7. For the stool samples of the upper row: first aspirate 250 μl of the ethanol-stool suspension into the 5 ml Eppendorf tube, then aspirate 250 μl of the ethanol-stool suspension into a corresponding Eppendorf tube for individual DNA extraction.
8. After pooling the ten samples into the 5 ml tube, close the tube and vortex well.
9. Aspirate two aliquots of 250 μl of the pooled sample for DNA extraction, and label these duplicates with the pool ID + ‘A’ or ‘B’.
   **Remark**: *We will extract the DNA of two aliquots of each pool and determine the*

*quantities of the different STHs using qPCR to document the reproducibility.*

10. Stop timing and fill in the time in RF10.

*Making pools of 20 and 60.*

1. For every site (BR, ET, LA or TA) and every visit (BL or FU), we will make all the pools of 20 and 60 in one time.
2. Start timing.
3. To make pools of 20 (P20), aspirate 1.5 ml of each of two pools of ten according to RF 10, add to a 5 ml tube and label. See also Figure 3 below.
4. Aspirate two aliquots of 250 µl of the pooled sample of 20 for DNA extraction, and label them with the pool ID + 'A' or 'B'.
   **Remark**: *We will extract the DNA of two aliquots of each pool and determine the quantities of the different STHs using qPCR to document the reproducibility.*
5. To make pools of 60 (P60), aspirate 1.5 ml of each of six pools of 60 according to RF 10, add to a 15 ml Falcon tube and label. See also Figure 3 below.
6. Aspirate two aliquots of 250 µl of the pooled sample of 60 for DNA extraction, and label them with the pool ID + 'A' or 'B'.
   **Remark**: *We will extract the DNA of two aliquots of each pool and determine the quantities of the different STHs using qPCR to document the reproducibility.*
7. Stop timing and fill in the time in RF 10.

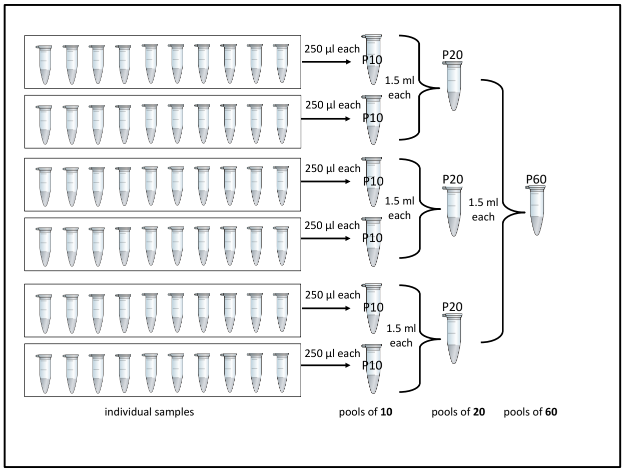


**Figure 3**. Pooling strategy.
