# Supplementary material for: Comprehensive evaluation of stool-based diagnostic methods and benzimidazole resistance markers to assess drug efficacy and detect the emergence of anthelmintic resistance: A Starworms study protocol
Source: PLoS Negl Trop Dis. 2018 Nov 2;12(11):e0006912. doi: 10.1371/journal.pntd.0006912 (PMC6235403; doi:10.1371/journal.pntd.0006912)
Supplement: S12 Info — (PDF) [file pntd.0006912.s012.pdf]

LF 01: Recruitment

Study: OPP1120972

Date of recruitment/ treatment: (DD/MMM/YYYY) __ / ___ / 2 0 1 7

Name of the school: ______________________

School ID (SXX): S _ _

Subject Identifier (XXXXX)	Name of the subject	Standard/ Grade (XX)	Age (XX)	Sex (M/F)	Initials interviewer (XXX)	Selected for Follow-Up *	Date of Follow-Up
B R _ _ _						☐ Yes ☐ No	__ / ___ / 2 0 1 7
B R _ _ _						☐ Yes ☐ No	__ / ___ / 2 0 1 7
B R _ _ _						☐ Yes ☐ No	__ / ___ / 2 0 1 7
B R _ _ _						☐ Yes ☐ No	__ / ___ / 2 0 1 7
B R _ _ _						☐ Yes ☐ No	__ / ___ / 2 0 1 7
B R _ _ _						☐ Yes ☐ No	__ / ___ / 2 0 1 7
B R _ _ _						☐ Yes ☐ No	__ / ___ / 2 0 1 7
B R _ _ _						☐ Yes ☐ No	__ / ___ / 2 0 1 7

* Mark YES if the subject tested positive for STH on any of the diagnostic test used. These subjects will need to be tested during follow-up visit.

Signature investigator: ______________________ **Date (DD/MMM/YYYY):** __ / ___ / ____

LF 02: Stool preservation

Study: OPP1120972

Subject ID (XXXXX)	Date of preservation (DD/MMM/YYYY)	Time in study	Initials of person preserving sample (XXX)	Comments
B R _ _ _	_ _ / _ _ _ / 2 0 1 7	☐ Baseline ☐ Follow-up	_ _ _	
B R _ _ _	_ _ / _ _ _ / 2 0 1 7	☐ Baseline ☐ Follow-up	_ _ _	
B R _ _ _	_ _ / _ _ _ / 2 0 1 7	☐ Baseline ☐ Follow-up	_ _ _	
B R _ _ _	_ _ / _ _ _ / 2 0 1 7	☐ Baseline ☐ Follow-up	_ _ _	
B R _ _ _	_ _ / _ _ _ / 2 0 1 7	☐ Baseline ☐ Follow-up	_ _ _	
B R _ _ _	_ _ / _ _ _ / 2 0 1 7	☐ Baseline ☐ Follow-up	_ _ _	
B R _ _ _	_ _ / _ _ _ / 2 0 1 7	☐ Baseline ☐ Follow-up	_ _ _	
B R _ _ _	_ _ / _ _ _ / 2 0 1 7	☐ Baseline ☐ Follow-up	_ _ _	
B R _ _ _	_ _ / _ _ _ / 2 0 1 7	☐ Baseline ☐ Follow-up	_ _ _	
B R _ _ _	_ _ / _ _ _ / 2 0 1 7	☐ Baseline ☐ Follow-up	_ _ _	

Signature investigator : ______________________________ **Date (DD/MMM/YYYY)** _ _ / _ _ _ / _ _ _ _

RF 01: Kato-Katz Preparation

Study: OPP1120972

☐ Baseline ☐ Follow up

Batch ID (XXXX)	Date of preparation (DD/MMM/YYYY)	Number of samples in batch (XX) (min 5, max 10)*	Time to prepare the slides (min:sec)	Initials of the lab technician (XXX)	Comments
B	/ / 2017		:		
B	/ / 2017		:		
B	/ / 2017		:		
B	/ / 2017		:		
B	/ / 2017		:		
B	/ / 2017		:		
B	/ / 2017		:		
B	/ / 2017		:		
B	/ / 2017		:		

* The goal is to process batches of 10 samples. For batches that consist out of less than 5 samples no timing procedures are required.

Signature investigator: ______________________ **Date (DD/MMM/YYYY):** / /

RF 02: Kato-Katz Examination

Study: OPP1120972

Date of examination (DD/MMM/YYYY): __ __ / __ __ __ / 2 0 1 7 ☐ Baseline ☐ Follow up

Subject ID (LAXXX)	Slide A/B	Number of *Ascaris* eggs (XXXX)	Number of *Trichuris* eggs (XXX)	Number of Hookworm eggs (XXX)	Time to read slide (min:sec)	Initials of the examiner (XXX)	Selected for stool preservation? *
B R	A				:		☐ Yes ☐ No
	B				:		
B R	A				:		☐ Yes ☐ No
	B				:		
B R	A				:		☐ Yes ☐ No
	B				:		
B R	A				:		☐ Yes ☐ No
	B				:		
B R	A				:		☐ Yes ☐ No
	B				:		

* During baseline examination, only stool samples found to contain at least 13 eggs on duplicate Kato-Katz or at least 15 eggs on Mini-FLOTAC for at least one of the three STH need to be preserved. During follow-up examination, all samples need to be conserved (See SOP 11).

Signature investigator: ______________________ **Date (DD/MMM/YYYY)** __ __ / __ __ __ / 2 0 1 7

RF 03: Kato Katz Quality Control

starworms

Study: OPP1120972

Date of Quality Control (DD/MMM/YYYY): __ __ / __ __ __ / 2 0 1 7

Subject ID (XXXXX)	Slide	Number of *Ascaris* eggs (XXXX)			Number of *Trichuris* eggs (XXX)			Number of Hookworm eggs (XXX)			Initials of 2nd examiner (XXX)
		Quality control exam.	First exam.	Meeting quality criteria*	Quality control exam.	First exam.	Meeting quality criteria*	Quality control exam.	First exam.	Meeting quality criteria*	
B R _ _ _	A			☐ Yes ☐ No			☐ Yes ☐ No			☐ Yes ☐ No	
	B			☐ Yes ☐ No			☐ Yes ☐ No			☐ Yes ☐ No	
B R _ _ _	A			☐ Yes ☐ No			☐ Yes ☐ No			☐ Yes ☐ No	
	B			☐ Yes ☐ No			☐ Yes ☐ No			☐ Yes ☐ No	

Comments:

*No false negatives/positives; difference in egg counts: ≤10 eggs difference when the total number of eggs counted ≤100 eggs (not eggs per gram of stool, but number of eggs counted under the microscope) or ≤ 20% when more than 100 eggs are counted. If sample does not meet these quality criteria, please follow the protocol described in SOP 10.

Signature investigator : ______________________ **Date (DD/MMM/YYYY) :** __ __ / __ __ __ / 2 0 1 7

RF 04: Mini-FLOTAC preparation

Study: OPP1120972

☐ BL ☐ FU

Batch ID (BXXX)	Date of preparation (DD/MMM/YYYY)	Number of samples in batch (min 5 - max 10) (XX)	**SOP 07, Section 4.2:** Preparing a batch of stool samples		Comments
			Time (min:sec)	Initials of the lab technician (XXX)	
	/ / 2 0 1 7		:		
	/ / 2 0 1 7		:		
	/ / 2 0 1 7		:		
	/ / 2 0 1 7		:		
	/ / 2 0 1 7		:		
	/ / 2 0 1 7		:		
	/ / 2 0 1 7		:		
	/ / 2 0 1 7		:		

* The goal is to process batches of 10 samples. For batches that consist out of less than 5 samples no timing procedures are required.

Signature investigator: ______________________________ **Date (DD/MMM/YYYY):** / /

RF 05: Mini-FLOTAC examination

Study: OPP1120972

Date of examination (DD/MMM/YYYY): __ / ___ / 2017

☐ Baseline ☐ Follow up

Subject ID (XXXXX)	Number of *Ascaris* eggs (XXXX)	Number of *Trichuris* eggs (XXX)	Number of Hookworm eggs (XXX)	Time to read the Mini-FLOTAC device (min:sec)	Initials of the examiner (XXX)	Selected for stool preservation? *
B R				:		☐ Yes ☐ No
B R				:		☐ Yes ☐ No
B R				:		☐ Yes ☐ No
B R				:		☐ Yes ☐ No
B R				:		☐ Yes ☐ No
B R				:		☐ Yes ☐ No
B R				:		☐ Yes ☐ No
B R				:		☐ Yes ☐ No

*During baseline examination, only stool samples found to contain at least 13 eggs on duplicate Kato-Katz or at least 15 eggs on Mini-FLOTAC for at least one of the three STH need to be preserved. During follow-up examination, all samples need to be conserved (See SOP 11).

Signature investigator: ______________________ **Date (DD/MMM/YYYY):** __ / ___ / ____

RF 06: Mini-FLOTAC Quality Control

Study: OPP1120972

Date of Quality Control (DD/MMM/YYYY): __ / ___ / 2017

Subject ID (XXXXX)	Number of *Ascaris* eggs (XXXX)			Number of *Trichuris* eggs (XXX)			Number of Hookworm eggs (XXX)			Initials of the 2nd examiner (XXX)
	Quality control exam.	First exam.	Meeting quality criteria*	Quality control exam.	First exam.	Meeting quality criteria*	Quality control exam.	First exam.	Meeting quality criteria*	
B R			☐ Yes ☐ No			☐ Yes ☐ No			☐ Yes ☐ No	
B R			☐ Yes ☐ No			☐ Yes ☐ No			☐ Yes ☐ No	
B R			☐ Yes ☐ No			☐ Yes ☐ No			☐ Yes ☐ No	
B R			☐ Yes ☐ No			☐ Yes ☐ No			☐ Yes ☐ No	
Comments:										

*No false negatives/positives; difference in egg counts: ≤10 eggs difference when the total number of eggs counted ≤100 eggs (not eggs per gram of stool, but number of eggs counted under the microscope) or ≤ 20% when more than 100 eggs are counted. If sample does not meet these quality criteria, please follow the protocol described in SOP 10.

Signature investigator: ______________________ **Date (DD/MMM/YYYY)** __ / ___ / ____

RF 07: FECPAK^{G2} Preparation

star worms

Study: OPP1120972

Date of preparation (DD/MMM/YYYY): ☐☐ / ☐☐☐ / ☐☐☐☐

Batch ID (BXXX)	Number of samples (XX) (min 5, max 10)*	**A) Preparation of the samples (Day 1):** Mix 3g of stool with water in Fill-FLOTAC, transfer to sedimenter, add water to the "water"-line, close the sedimenter, invert 3 times to mix sample and put aside for overnight sedimentation. **(SOP 08, section 4.1.1).**		**B) Completing submission forms.** Create one submission per batch and add the number of samples to be processed to the sample list. **(SOP 08, section 4.1.2)**		Comments
		**Time (min:sec)**	**Initials of the lab technician (XXX)**	**Time (min:sec)**	**Initials of the lab technician (XXX)**	
B☐☐☐	☐☐	☐☐ : ☐☐	☐☐☐	☐☐ : ☐☐	☐☐☐	
B☐☐☐	☐☐	☐☐ : ☐☐	☐☐☐	☐☐ : ☐☐	☐☐☐	
B☐☐☐	☐☐	☐☐ : ☐☐	☐☐☐	☐☐ : ☐☐	☐☐☐	
B☐☐☐	☐☐	☐☐ : ☐☐	☐☐☐	☐☐ : ☐☐	☐☐☐	

* The goal is to process batches of 10 samples. For batches that consist out of less than 5 samples no timing procedures are required

Signature investigator: ______________________________ **Date (DD/MMM/YYYY):** ☐☐ / ☐☐☐ / ☐☐☐☐

RF 07: FECPAK^{G2} Preparation

starworms

Study: OPP1120972

Date of preparation (DD/MMM/YYYY): ☐☐ / ☐☐☐ / ☐☐☐☐

**Batch ID (BXXX)**	**Number of samples (XX)** (min 5, max 10)*	**C) Preparation of the cassettes (Day 2):** Decant sedimenters, add saline to "saline"-line, transfer to cylinder and insert sieves. Invert 3 times and fill first well of the cassette. Invert cylinder again 3 times and fill second well of cassette. **(SOP 08, section 4.2.1).**		**D) Image capture.** Initiate software, select correct sample, click add images, insert cassette into the Micro-I and follow the instructions on the screen. **(SOP 08, section 4.2.2).**		**Comments**
		**Time (min:sec)**	**Initials of the lab technician (XXX)**	**Time (min:sec)**	**Initials of the lab technician (XXX)**	
B☐☐☐	☐☐	☐☐ : ☐☐	☐☐☐	☐☐ : ☐☐	☐☐☐	
B☐☐☐	☐☐	☐☐ : ☐☐	☐☐☐	☐☐ : ☐☐	☐☐☐	
B☐☐☐	☐☐	☐☐ : ☐☐	☐☐☐	☐☐ : ☐☐	☐☐☐	
B☐☐☐	☐☐	☐☐ : ☐☐	☐☐☐	☐☐ : ☐☐	☐☐☐	

* The goal is to process batches of 10 samples. For batches that consist out of less than 5 samples no timing procedures are required.

Signature investigator: ______________________________ **Date (DD/MMM/YYYY):** ☐☐ / ☐☐☐ / ☐☐☐☐

RF 08: FECPAK^{G2} examination

Study: OPP1120972

Date of examination (DD/MMM/YYYY): __ / ___ / 2 0 1 7

☐ Baseline ☐ Followup

SAMPLE ID (XXXXXX)	Number of *Ascaris* eggs (XXXX)	Number of *Trichuris* eggs (XXX)	Number of Hookworm eggs (XXX)	Time needed for markup (min:sec)	Initials of the examiner (XXX)	Comments
				:		
				:		
				:		
				:		
				:		
				:		
				:		
				:		
				:		
				:		

Signature investigator: ______________________________ **Date (DD/MMM/YYYY)** __ / ___ / 2 0 1 7

RF 09: FECPAK^{G2} Quality Control

star worms

Study: OPP1120972

Date of quality control (DD/MMM/YYYY): ☐☐ / ☐☐☐ / ☐☐☐☐

U-Ghent use only

Subject ID (XXXXX)	Number of *Ascaris* eggs (XXXX)			Number of *Trichuris* eggs (XXX)			Number of Hookworm eggs (XXX)			Initials of the 2nd examiner (XXX)
	Quality control exam.	First exam.	Meeting quality criteria*	Quality control exam.	First exam.	Meeting quality criteria*	Quality control exam.	First exam.	Meeting quality criteria*	
☐☐☐☐☐	☐☐☐☐	☐☐☐☐	☐ Yes<br>☐ No	☐☐☐	☐☐☐	☐ Yes<br>☐ No	☐☐☐	☐☐☐	☐ Yes<br>☐ No	☐☐☐
☐☐☐☐☐	☐☐☐☐	☐☐☐☐	☐ Yes<br>☐ No	☐☐☐	☐☐☐	☐ Yes<br>☐ No	☐☐☐	☐☐☐	☐ Yes<br>☐ No	☐☐☐
☐☐☐☐☐	☐☐☐☐	☐☐☐☐	☐ Yes<br>☐ No	☐☐☐	☐☐☐	☐ Yes<br>☐ No	☐☐☐	☐☐☐	☐ Yes<br>☐ No	☐☐☐
☐☐☐☐☐	☐☐☐☐	☐☐☐☐	☐ Yes<br>☐ No	☐☐☐	☐☐☐	☐ Yes<br>☐ No	☐☐☐	☐☐☐	☐ Yes<br>☐ No	☐☐☐
Comments:										

*No false negatives/positives; difference in egg counts: $\leq$ 10 eggs difference when the total number of eggs counted $\leq$ 100 eggs (not eggs per gram of stool, but number of eggs counted under the microscope) or $\leq$ 20% when more than 100 eggs are counted. If sample does not meet these quality criteria, please follow the protocol described in SOP 10.

Signature investigator: ______________________ **Date (DD/MMM/YYYY)** ☐☐ / ☐☐☐ / ☐☐☐☐

TRF 01: Data entry of baseline demographics

starworms

Study: OPP1120972

Data entry ID (DXXX)	Date of data entry (DD/MMM/YYYY)	Number of samples for which data was entered (min 10 –max 50) (XX)	Time needed to enter baseline demographics data (hh:min:sec)	Initials of person entering the data (XXX)	Comments
D	/ / 2017		: :		
D	/ / 2017		: :		
D	/ / 2017		: :		
D	/ / 2017		: :		
D	/ / 2017		: :		
D	/ / 2017		: :		
D	/ / 2017		: :		
D	/ / 2017				
D	/ / 2017		: :		
D	/ / 2017		: :		

Signature investigator : ________________________________ **Date (DD/MMM/YYYY)** / /

TRF 02: Data entry of Kato-Katz results

Study: OPP1120972

Data entry ID (DXXX)	Date of data entry (DD/MMM/YYYY)	The number of samples for which data was entered (min10 – max 50) (XX)	Baseline (BL) or Follow-up (FU) data?	Time to enter Kato-Katz results (hh:min:sec)	Initials of person entering data (XXX)	Comments
D	/ / 2017		BL FU	: :		
D	/ / 2017		BL FU	: :		
D	/ / 2017		BL FU	: :		
D	/ / 2017		BL FU	: :		
D	/ / 2017		BL FU	: :		
D	/ / 2017		BL FU	: :		
D	/ / 2017		BL FU	: :		
D	/ / 2017		BL FU	: :		
D	/ / 2017		BL FU	: :		
D	/ / 2017		BL FU	: :		

Signature investigator: ______________________________ **Date (DD/MMM/YYYY)** / /

TRF 03: Data analysis and reporting

Study: OPP1120972

Data analysis / reporting ID (AXX)	Date of data analysis or reporting of results (DD/MMM/YYYY)	Time to analyse data or report results (hh:min:sec)	Initials of person performing task (XXX)	Task Performed. (See SOP 14 for more info on data analysis and reporting)
A	/ /	: :		
A	/ /	: :		
A	/ /	: :		
A	/ /	: :		
A	/ /	: :		
A	/ /	: :		
A	/ /	: :		

Comments:

Signature investigator: ______________________________ **Date (DD/MMM/YYYY)** / /
