# Supplementary material for: Comprehensive evaluation of stool-based diagnostic methods and benzimidazole resistance markers to assess drug efficacy and detect the emergence of anthelmintic resistance: A Starworms study protocol
Source: PLoS Negl Trop Dis. 2018 Nov 2;12(11):e0006912. doi: 10.1371/journal.pntd.0006912 (PMC6235403; doi:10.1371/journal.pntd.0006912)
Supplement: S16 Info — (PDF) [file pntd.0006912.s016.pdf]

Comparison of Double Entered Data

1. Purpose

In the Starworms study, all raw data are entered into excel files, the Data Entry Files (DEFs), using double data entry policy, i.e. all data are entered by two independent data entry clerks.

This SOP describes the procedures to identify discrepancies between the different DEFs that have been generated during the double data entry process.

2. Equipment

- Personal Computer or laptop with Excel

3. Forms and files

RF 01, RF 02, RF 04, RF 05, RF 07, RF 08	
DEF 01_PersonA, DEF01_PersonB, DEF 03_PersonA and DEF03_PersonB	

4. Procedures

4.1. Installation of the 'Inquire' add-in in Excel

1. Open Excel, click on 'options' under 'file'. A new window opens.
2. On the left hand side, choose 'Add-ins'.

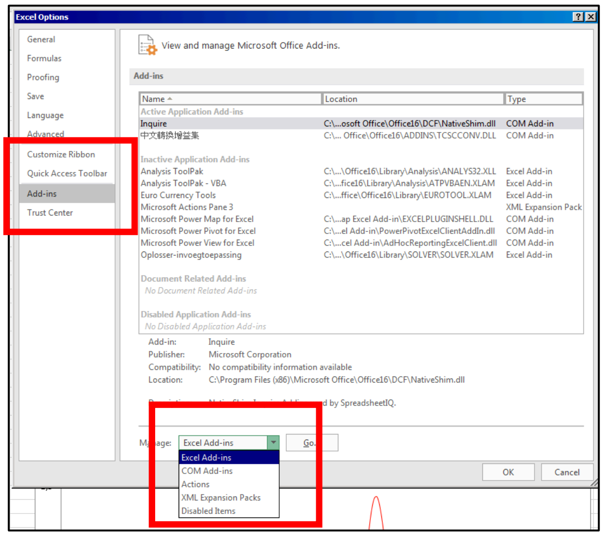

3. In the 'Manage' dropdown menu, select 'COM Add-Ins' and click on 'Go'.

4. Thick 'Inquire' and click on 'OK'.

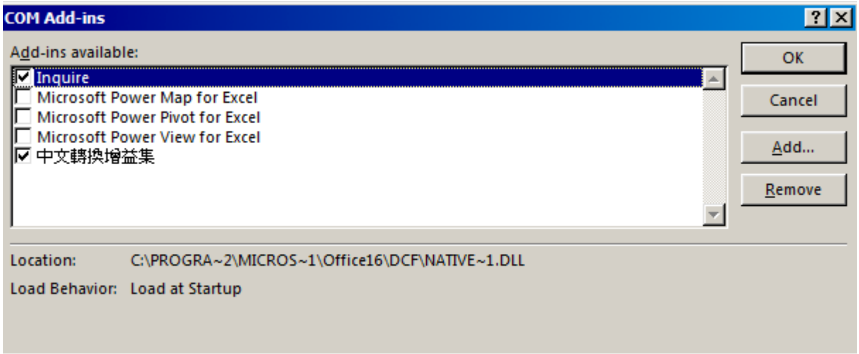

4.2. Comparing two DEFs.

1. Open the two files that you want to compare for inconsistencies, e.g. DEF01_PersonA_20160901 and DEF01_PersonB_20160901.

2. Choose 'Compare Files' under the Inquire add-in: a dropdown list will show you all opened files. Choose the two files that you want to compare and click on 'Compare'.

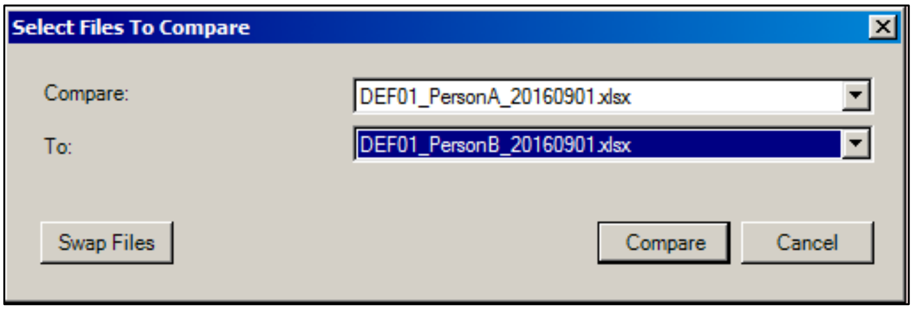

3. A new document, divided in 5 parts, opens:

(i) above, left, you see the data from one DEF

(ii) above, right, you see the data from the other DEF

(iii) below, in the middle, you see the list with all inconsistencies between the two files

(iv) below, left, you see the color code explaining the type of inconsistencies

(v) below, right, you see some statistics.

In the title bar, you see which DEF is on the left and the right.

The inconsistencies are marked throughout the document. In the example below, two inconsistencies are shown: *Trichuris* counts of Kato-Katz slide A for TA001 and *Trichuris* counts of Kato-Katz slide B for TA010.

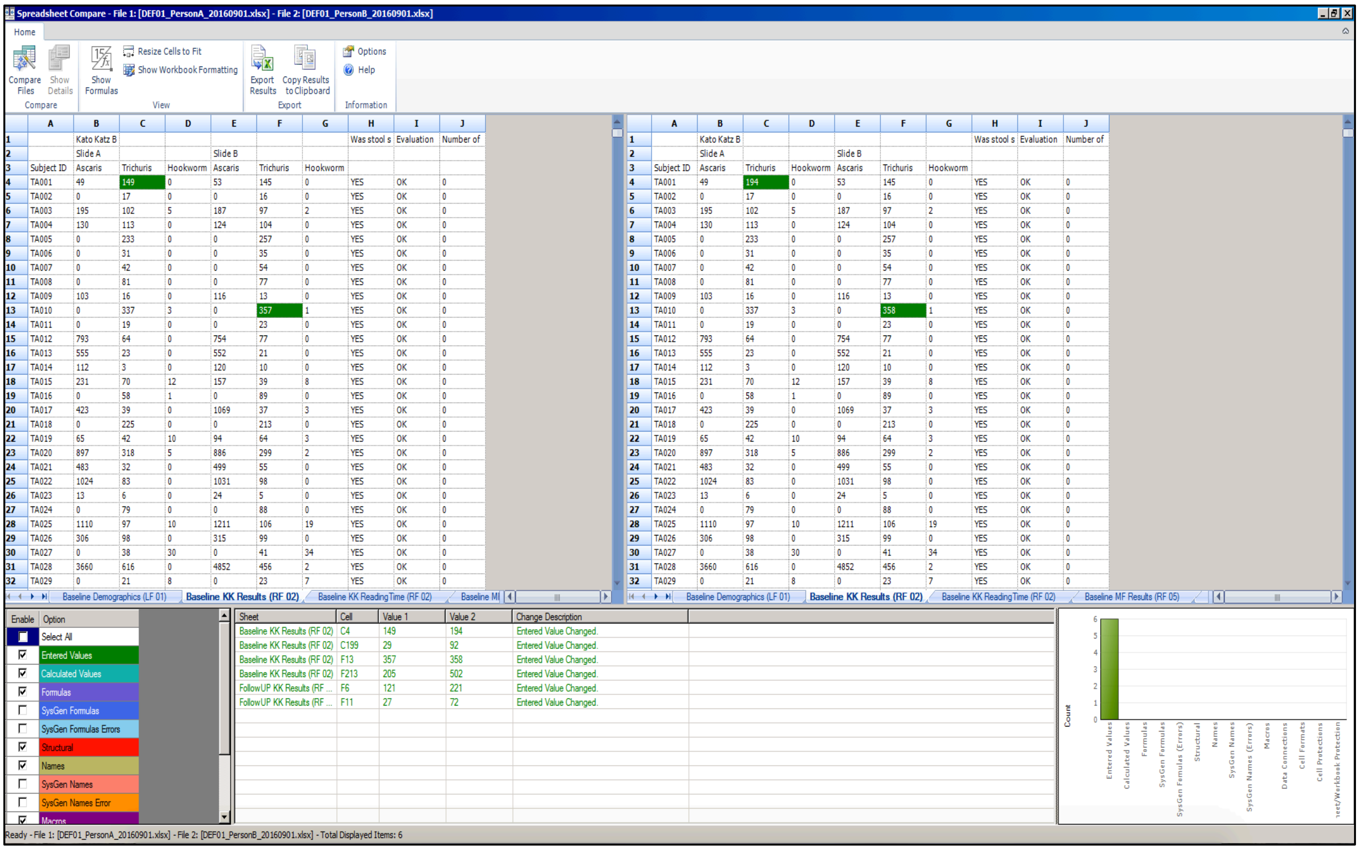

4. Save one of the DEFs as 'DEF01_**PersonC_QC**'.

5. Identify all inconsistencies between the 2 DEFs, check the correct values in the raw data, i.e. the different Record Forms, and correct in the 'DEF01_PersonC_QC' if necessary.
